# The Contribution of Teleconsultation and Videoconferencing to Diabetes Care: A Systematic Literature Review

**DOI:** 10.2196/jmir.9.5.e37

**Published:** 2007-12-14

**Authors:** Fenne Verhoeven, Lisette van Gemert-Pijnen, Karin Dijkstra, Nicol Nijland, Erwin Seydel, Michaël Steehouder

**Affiliations:** ^3^Department of Marketing Communication and Consumer PsychologyFaculty of Behavioral SciencesUniversity of TwenteEnschedeThe Netherlands; ^2^Department of Psychology and Communication of HealthFaculty of Behavioral SciencesUniversity of TwenteEnschedeThe Netherlands; ^1^Department of Technical and Professional CommunicationFaculty of Behavioral SciencesUniversity of TwenteEnschedeThe Netherlands

**Keywords:** Chronic diseases, diabetes mellitus, telemedicine, consultation, teleconsultation, videoconferencing

## Abstract

**Background:**

A systematic literature review was carried out to study the benefits of teleconsultation and videoconferencing on the multifaceted process of diabetes care. Previous reviews focused primarily on usability of technology and considered mainly one-sided interventions.

**Objective:**

The objective was to determine the benefits and deficiencies of teleconsultation and videoconferencing regarding clinical, behavioral, and care coordination outcomes of diabetes care.

**Methods:**

Electronic databases (Medline, PiCarta, PsycINFO, ScienceDirect, Telemedicine Information Exchange, ISI Web of Science, Google Scholar) were searched for relevant publications. The contribution to diabetes care was examined for clinical outcomes (eg, HbA_1c_, blood pressure, quality of life), behavioral outcomes (patient-caregiver interaction, self-care), and care coordination outcomes (usability of technology, cost-effectiveness, transparency of guidelines, equity of care access). Randomized controlled trials (RCTs) with HbA_1c_ as an outcome were pooled using standard meta-analytical methods.

**Results:**

Of 852 publications identified, 39 met the inclusion criteria for electronic communication between (groups of) caregivers and patients with type 1, type 2, or gestational diabetes. Studies that evaluated teleconsultation or videoconferencing not particularly aimed at diabetes were excluded, as were those that described interventions aimed solely at clinical improvements (eg, HbA_1c_). There were 22 interventions related to teleconsultation, 13 to videoconferencing, and 4 to combined teleconsultation and videoconferencing. The heterogeneous nature of the identified videoconferencing studies did not permit a formal meta-analysis. Pooled results from the six RCTs of the identified teleconsultation studies did not show a significant reduction in HbA_1c_ (0.03%, 95% CI = - 0.31% to 0.24%) compared to usual care. There was no significant statistical heterogeneity among the pooled RCTs (*χ* ^2^_7_= 7.99, *P* = .33). It can be concluded that in the period under review (1994-2006) 39 studies had a scope broader than clinical outcomes and involved interventions allowing patient-caregiver interaction. Most of the reported improvements concerned satisfaction with technology (26/39 studies), improved metabolic control (21/39), and cost reductions (16/39). Improvements in quality of life (6/39 studies), transparency (5/39), and better access to care (4/39) were hardly observed. Teleconsultation programs involving daily monitoring of clinical data, education, and personal feedback proved to be most successful in realizing behavioral change and reducing costs. The benefits of videoconferencing were mainly related to its effects on socioeconomic factors such as education and cost reduction, but also on monitoring disease. Additionally, videoconferencing seemed to maintain quality of care while producing cost savings.

**Conclusions:**

The selected studies suggest that both teleconsultation and videoconferencing are practical, cost-effective, and reliable ways of delivering a worthwhile health care service to diabetics. However, the diversity in study design and reported findings makes a strong conclusion premature. To further the contribution of technology to diabetes care, interactive systems should be developed that integrate monitoring and personalized feedback functions.

## Introduction

Diabetes mellitus (DM) is major chronic disease that demands teamwork from various caregivers for the delivery of high-quality care [1]. Consequently, an adequate communication structure is an important condition for optimal interaction and coordination among caregivers and between patients and caregivers [2]. Information and communication technology (ICT) is often seen as the solution for problems in the management of diabetes care because of its potential to enhance care coordination and support patient self-care [3]. It is expected that using ICT will reduce costs while maintaining high-quality health care and that ICT can respond to an increasing demand for care with a decreasing availability of personnel [4]. Previous reviews on diabetes care have found modest benefits of ICT-based care compared to conventional face-to-face care. However, these reviews focused primarily on the usability of technology and considered mainly one-sided interventions such as clinical improvements (glucose and diet) rather than looking at the multifaceted process of a diabetic patient, including relevant issues such as the influence of interactive technology on the process of care (patient-caregiver collaboration, care coordination, costs) and patient outcomes such as quality of life and self-care [2,4,5].

ICT-based care is more than just a technological intervention—it includes a way of thinking about how to deliver health care with the aid of ICT [6]. The most important modalities of ICT-based care are teleconsultation and videoconferencing [1]. Teleconsultation is a kind of telemonitoring including patient-caregiver communication (monitoring and delivering feedback) via email, phone, automated messaging systems, other equipment without face-to-face contact, or the Internet [7]. Videoconferencing involves real-time face-to-face contact (image and voice) via videoconferencing equipment (television, digital camera, videophone, etc) to connect caregivers and one or more patients simultaneously, usually for instruction [8].

The aim of this review is to obtain an overview of the existing empirical support for the alleged benefits of teleconsultation and videoconferencing on diabetes care. The benefits are evaluated by means of criteria for “good chronic care” [1,2]. The evaluation criteria are clinical outcomes, behavioral outcomes, and care coordination outcomes [1,2]. Clinical outcomes include metabolic control and of life. Behavioral outcomes include self-care and patient-caregiver interaction. Care coordination outcomes refer to cost-effectiveness, transparency of the care delivery process, equity of access to care, and usability of equipment to facilitate the care delivery process.

Our review is intended to inform social scientists and practitioners about the potential of technology to improve diabetes care. We provide an overview of what is currently known about the benefits and deficits of teleconsultation and videoconferencing and how practical and worthwhile these services are [9].

## Methods

### Literature Search

We collected publications (from May 2005 to December 2007) on empirical research on ICT-based interaction between caregivers and patients or groups of patients, or among caregivers or patients themselves, using the for systematic reviews developed by the Centre for Reviews and Dissemination [10]. The review was restricted to studies evaluating teleconsultation and/or videoconferencing developed for type 1, type 2, and/or gestational diabetes and to language publications published between 1994 and 2006.

No restrictions were imposed on the quality of study design because assessment studies dealing with ICT-based care are scarce [9], and, in practice, reviews have been constrained by the availability of data. In particular, behavioral or care coordination aspects were seldom the focus of reviews on diabetes care. Most reviews focused solely on clinical values in randomized controlled trials (RCTs). In light of a holistic approach, we wanted to provide a broad range of information in order to facilitate decisions about implementing new technology in health care. We excluded studies dealing with broader target groups than diabetics, studies not aimed at patient-caregiver interaction but solely reporting technical aspects of the equipment used, and those that strived for clinical improvements only. We included studies that covered clinical outcomes plus one or more other outcomes (behavioral, care coordination).

The following electronic databases on medicine, psychology, and telemedicine were searched: Medline, ScienceDirect, ISI Web of Science, Telemedicine Information Exchange, PsycINFO, PiCarta, Google Scholar, and journal indexes (Diabetes Care, Effective Health Care, Journal of Medical Internet Research, Journal of Medical Informatics, Telemedicine and E-health, Telemedicine and Telecare). Keyword sets combined “diabetes” and one of the following: “telemedicine,” “telecare,” “telehealth,” “e-health,” “teleconsultation,” “telemonitoring,” or “videoconferencing.” We used “telemedicine” because the terms “e-health” and “electronic care” were hardly used in the literature before 2004. In addition to the databases, the reference lists of the identified publications were hand-searched. Citation was reviewed and designated as “in,” “out,” or “uncertain” based on the aforementioned restrictions. Sources designated as “in” or “uncertain” were obtained for further review. Two of the authors independently reviewed titles and abstracts of the identified publications to decide whether they should be examined in full detail.

Two authors completed data extraction forms developed by the Centre for Reviews and Dissemination [10] and recorded the following details: study design (evidence level and methods for measurement outcomes, patient selection, description of intervention and control groups), study population (type of diabetes, age group, number and recruitment of patients), and intervention details (care setting, technology used to support the care delivery process, duration of the intervention). Using the care levels previously mentioned (clinical, behavioral, and care coordination) [1,2], we developed a checklist to categorize the outcomes of the interventions (Table 1).

**Table 1:** Checklist to classify the outcome measures related to the levels of diabetes care

Level of Diabetes Care	Outcome Measures
Clinical	Improved clinical values (eg, dietary values, HbA_1c_ , blood pressure) Improved quality of life (social functioning, general or mental health, well-being, and satisfaction with care)
Behavioral	Improved interaction (communication between caregivers and patients or among caregivers or patients themselves) Improved self-care (ablity to control diabetes and to cope with diabetes via self-monitoring, education, knowledge about diabetes, and personal feedback)
Care coordination	Improved usability (and adoption) of technology Reduction of costs (saving patients’ or caregivers’ time and reducing the use of health care services) Improved transparency (care delivery based on standards as guidelines, protocols for information exchange) Improved equity (the availability of health care to everyone)

Five levels [10] were used to categorize the methodological approaches of the studies (Table 2). Two authors independently rated the study designs. In case of disagreement, consensus was reached by discussion.

**Table 2:** Checklist to categorize level of evidence of study design

Level of Evidence	Study Design
1	Experimental studies (eg, RCT with blinded allocation)
2	Quasi-experimental studies (eg, experimental study with randomization)
3	Controlled observational studies
3a	Cohort studies
3b	Case control studies
4	Observational studies without control groups
5	Expert opinion based on bench research or consensus

### Statistical Methods

A quality assessment was completed for all RCTs using the Jadad scale [11]. This scale contains questions about randomization, blinding, and withdrawals that are scored by a yes (1) or no (0). In total, five points can be awarded, with higher points indicating higher study quality.

Changes in HbA_1c_ values were calculated from baseline and follow-up means and standard deviations. Only studies researching effects on adults were included in the meta-analysis. When the deviation of the mean difference was not available in the papers, the authors were contacted. In case of no response or no availability of the requested information, we estimated the variance by using (1) reported confidence intervals, (2) reported *P* values, or (3) an imputation technique [12]. A random-effects model was used for pooling the included studies because clinical heterogeneity between studies was expected. The between-study heterogeneity was tested using the chi-square statistic. In one study, three intervention groups and one control group were studied. In meta-analyses, all three intervention groups were compared with the same usual care group, resulting in two extra comparisons.

## Results

### Study Characteristics

**Figure 1.**
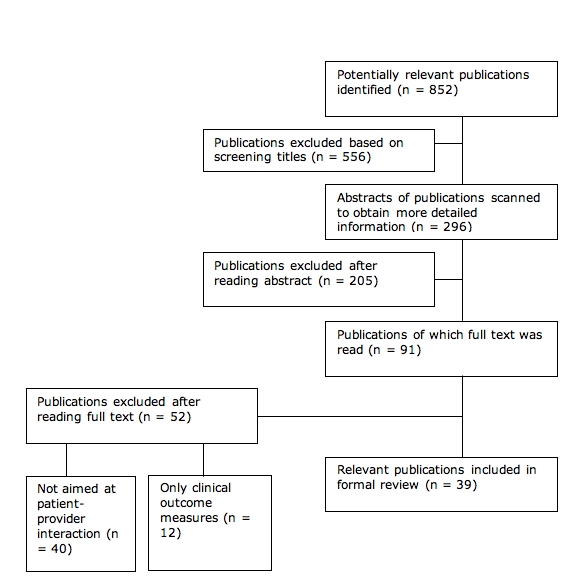
Study selection process

We identified 852 potentially relevant publications, 39 of which were included after the selection procedure described in the previous section (Figure 1).

Table 3 (teleconsultation) and Table 4 (videoconferencing) summarize the characteristics of the publications that were included. As can be seen, 22 interventions addressed teleconsultation [13-34], 13 addressed videoconferencing [35-47], and 4 addressed videoconferencing combined with teleconsultation [48-51]. The most frequently used methodological approach was observational (case series or before-after design), which was used in 19 studies; 11 studies were RCTs, and 6 were quasi-experimental. The other methodological approaches were used only incidentally (two cohort studies and one study based on expert interviews). Sample sizes included ≤ 20 (n = 9), ≤ 100 (n = 17), > 100 (n = 12), and one was not specified. Participants were selected by the research team [31,33,36,47,49,50], general practitioner [17,30,35,47], a specialist [15,17,22,24], or via convenience sampling [32,40].

Data were gathered via interviews, focus groups, log files, and nonstandardized questionnaires. Validated questionnaires were used in 12 of the 39 studies to measure usability of technology, quality of life, and self-care. The Telemedicine Satisfaction Questionnaire [14,25,41,47] was used for measuring usability of technology. Quality of life was measured with various questionnaires: the World Health Organization Quality of Life-BREF [14], SF-12 [17,25,30], SF-36 [36,38,41,44,48,49], Diabetes Quality of Life [25,30,36,41,44], Depression Scale CES-D [30], Problem Areas in Diabetes Scale [41], and the Visual Analog Scale [26]. The Diabetes Knowledge Assessment [36], Diabetes Treatment Satisfaction Questionnaire [41], and the Appraisal of Diabetes Scale [41] were used to measure self-care.

Table 3 and Table 4 present the improvements found in the studies, per outcome (clinical values, quality of life, patient-caregiver interaction, self-care, usability, cost reduction, transparency, and equity). Most of the studies reported improvements in usability of technology (n = 26; 15 teleconsultation and 11 videoconferencing), followed by clinical improvements (n = 21; 15 teleconsultation and 6 videoconferencing), cost reduction (n = 16; 5 teleconsultation and 11 videoconferencing), self-care (n = 14; 10 teleconsultation and 4 videoconferencing), and patient-caregiver interaction (n = 13; 10 teleconsultation and 3 videoconferencing). A minority of the studies reported improvements in quality of life (n = 6, 3 teleconsultation and 3 videoconferencing), transparency of care delivery guidelines (n = 5, 3 teleconsultation and 2 videoconferencing), and equity in access to health care (n = 4; all videoconferencing).

The findings summarized in Table 3 and Table 4 were extracted from publications that varied in study design and data gathering methods, and the reported findings were often not substantiated with evidence, as can be seen in the tables. In the light of the purpose of our review, we took this heterogeneity in study characteristics into account.

To get insight into the contribution of teleconsultation and videoconferencing to diabetes care, the results of these interventions were presented separately, describing care setting and intervention and clinical, behavioral, and care coordination outcomes. Improvements were reported and explained.

**Table 3:** Overview of teleconsultation interventions (see Multimedia Appendix for full tables containing inclusion criteria and data gathering methods)

Reference, Country, Year, and Duration of Intervention	Care Setting and Intervention	Study Design	Reported Findings (Improvements) ^*^
[13] Italy/Spain/Norway 2002 18 months, follow-up planned (duration unknown)	Secondary care. Blood glucose meter to send clinical data and lifestyle data (every 7 to 10 days) via telecommunication system (Internet / telephone line). Daily computer-generated feedback is provided, and, if necessary, messages from physician (specialist in hospital) to advise patients. No details provided about feedback system and frequency of feedback.	Observational studies without control group: n = 32 Four conditions: Verification phase: clinical evaluation (n = 3) Pilot clinical validation (n = 12) Demonstration phase: Intranet (n = 6) Demonstration phase: Internet (n = 11)
[14] Italy/Spain/Germany 2003 12 months, follow-up unknown	Integrated care. Reflectometer and palmtop to transmit clinical data via multi-access system (Web access, telephone, interactive voice) to each agent involved in the care process: nurses, case managers, and specialists. Computer-generated feedback is provided via SMS or email to patient and caregiver and educational messages are automatically sent to patients. Frequency of feedback not specified.	Experimental studies (RCT): n = 106 Two conditions: Intervention: n = 56 (subset randomized patients not reported) Control (usual care): n = 50 (subset randomized not reported) Randomization method not described; no details about comparability of group (except same clinical treatment)	a) Decreased HbA_1c_ in (I: 8.31 to 7.59, *P* < .05; C: 8.86 to 7.95, *P* < .05 after 6 months). NSD between groups. Patients randomized decreased HbA_1c_ (I: 8.24 to 7.44, *P* < .05; C: 8.83 to 7.78, *P* < .05, after 6 months). NSD.
[15] Germany 2002 4-8 months, follow-up unknown	Secondary care. Blood glucose meter to send clinical data via modem and telephone line to physician in diabetes center. Personal feedback for proper dose adjustment by diabetes specialist via telephone advice. Frequency of feedback not specified.	Experimental studies (RCT): n = 43 Two conditions: Intervention: n = 27 Control (usual care): n = 16 Randomization by lots (2:1 in favor of telecare). Fairly good matching of groups	a) Decreased HbA_1c_ (I: 8.3 to 6.9 after 4 months, n = 27; to 7.1 after 8 months, n = 11; C: 8.0 to 7.0 after 4 months, n = 16, to 6.8 after 8 months, n = 10). NSD between groups. e) System appeared easy to use; patients’ feeling of security increased through availability of BG data and the possibility of consulting a caregiver within minimal time, without the need to travel to the diabetes center. f) Cost and time savings in I (saving in consultation time although intensified contacts with caregiver); on caregiver’s side patient’s time significantly increased.
[16] Netherlands 1999 12 months, follow-up unknown	Primary/secondary care. Electronic communication network linking the physicians’ computer-based patient records (GPs and interns in hospital) to enable electronic data interchange. System provides computer-generated prompts for physicians to deliver feedback (messages). Frequency of feedback not specified.	Quasi-experimental studies: n = 275 Two conditions: Intervention: n = 215 Control (usual care): n = 60 Intervention group consisted of patients from GPs with highest number of referred patients. Average age in intervention group higher; fewer type 1 patients than control group -Nonstandardized questionnaire	a) Decreased HbA_1c_ (I: 7.0 to 6.8, *P* < .05; C: 6.6 to 6.5, *P* = .52). NSD between groups. c) Increased frequency of patient-caregiver communication (*P* < .01); more complete information about patient care in I than in C.
[17] United States 2002 12 months, follow-up unknown	Secondary care. Health Hero iCare Desktop and Health Buddy appliance for daily monitoring of clinical data and educational reinforcement by case manager (profession not specified) in medical center. System prompts to action if indicated by daily values. Personal feedback by telephone in case of alarming values.	Controlled observational studies (cohort studies): n = 338 Two conditions: Intervention: n = 169 Control: n = 169 (cohort) Cohort representative of the general population in terms of ethnicity	b) Mean improvement in mental component (SF-12) after 6 months in I (*P* < .0264) and in physical component after 6 months (*P* < .0518). c) Increased satisfaction regarding communication with caregivers in I (from 88% of patients after 3 months to 95% at 1 year). d) Better understanding of their medical condition (93% of patients), better able to manage their disease (93% of patients) after 1 year. e) Ease of use increased over time (75% of patients after 3 months to 88% after 1 year). f) Reduction of overall utilization and charges after 1 year; in I overall charges of US $747 per patient per year; inpatient admissions reduced 32% (*P* < .07); emergency room encounters reduced 34% (*P* < .06); post-discharge care visits reduced 44% (*P* < .028); outpatient visits reduced 49% (*P* < .001).
[18] Denmark 2006 6 months, follow-up unknown	Integrated care. Website for transmission of blood glucose data entered by patient and reviewed by diabetes team (2 diabetic nurses, 1 consultant doctor, 1 medical secretary, and 1 dietitian) and personal feedback by diabetes team by email about diabetes regimen. Frequency of feedback not specified. Based on theory of patient-centeredness and the Bayesian model of carbohydrate metabolism.	Observational studies without control group (case series): n = 13 Three conditions: Patients: n = 3 Health care professionals: n = 5 Health care professionals: n = 5 (focus group)	c) Patients experienced greater confidence and a more personal report with staff after 6 months using the system. Email facilitated a dialogue between patient and diabetes team. d) Improved self-control (patients checked blood glucose more often); increased awareness of blood sugar regulations. e) DiasNet caused changes in tasks and duties of the diabetes team (required enhanced competence of nurse with regard to insulin dose adjustments); patients were dissatisfied with the feedback from staff.
[19] United Kingdom 2005 9 months, follow-up unknown	Secondary care. Blood glucose monitor and telephone network for transmission of data and GPRS mobile phone to send data (daily) to diabetes nurse specialist in clinic. Real-time graphical phone-based feedback for the previous 2 weeks together with nurse-initiated support using a Web-based graphical analysis of glucose self-monitoring results and personal feedback by phone in case of concerns. Frequency of feedback not specified. Based on theory of patient-centeredness.	Experimental studies (RCT): n = 93 Two conditions: Intervention: n = 47 (Web-based graphical analysis, nurse initiated support) Control (real-time graphical phone-based): n = 46 Randomization (computer program); gender and psychiatric scores evenly distributed between the randomized groups	a) Decreased HbA_1c_ (I: 9.2 to 8.6 after 9 months, *P* < .001; C: 9.3 to 8.9 after 9 months, *P* < .05). NSD between groups. e) Difference in proportion of transmitted blood glucose results (40% more in I than in C, *P* < .0001).
[20] Spain 2004 9 months, follow-up unknown	Care setting not specified. PC, Web browser, or a cell phone with Wireless Application Protocol for transmission of clinical data. Automatic generated responses and personal feedback by physicians (not specified whether GPs or specialists in hospital) that could be read during patient’s next online session. Frequency of feedback not specified.	Observational studies without control group (case series): n = 172 Two conditions: Case study: n = 12 Questionnaire: n = 160 (135 non-diabetic students, 25 diabetic patients)	d) Patients were satisfied with the continuity and self-efficacy of care; lack of time was a drawback for 38%; 75% expressed a preference for sending data via a cellular phone (SMS). e) Patients used the system every 2.0 days (SD 2.1), and doctors reviewed patient data every 4.0 days (SD 3.9); the average number of visits to the website was 477 per month.
[21] Spain 2004 8 months, follow-up unknown	Care setting not specified. Patients send blood glucose levels and body weight to a server by SMS. Automatic server answers SMS each time data were sent. Monthly hemoglobin results automatically sent to physicians (not specified whether GPs or specialists in hospital). Physicians can send messages to patients if necessary.	Observational studies without control group (case series): n = 23 One condition	e) SMS provided a simple, fast, efficient, and low-cost adjunct to the medical management of diabetes at a distance. Particularly useful for age groups (elderly, teenagers) that are known to have difficulty in controlling diabetes well. f) Total of 25 messages per month; €3.75 per month per patient.
[22] France 2006 6 months, follow-up unknown	Secondary care. Clinical data from patients’ glucose meters are downloaded every 2 weeks to pharmacists’ PC. Reinforced follow-up via fax mediated by the local pharmacist in contact with the specialist in the hospital (diabetologist). Diabetologist sends instruction to family by email or phone within 5 days.	Experimental studies (RCT): n = 100 Two conditions: Intervention: n = 50 Control (usual care): n = 50 Randomization via computer-generated sequence using block randomization with stratification by age Comparable intervention and control groups (age, gender, HbA_1c_, frequency of SBGM, type of insulin therapy program)	a) Decreased HbA_1c_ (I: 9.3 to 9.27, *P* = .59; C: 9.2 to 9.12, *P* = .58). NSD between groups. c) Caregivers’ response to faxes was 81% (at 3 months); decreased to 50% (6 months). d) Frequency of self blood glucose monitoring per day did not differ between groups at the end of the study (*P* = .53). e) Only 32% of faxes (out of 100% expected) from family homes were received due to technical problems.
[23] Spain 2002 12 months, follow-up expected	Secondary care. Clinical data from a blood glucose meter are sent (automatically or manually) from a patient unit to the medical workstation for physicians (diabetologist in hospital). System offers tools to collect, manage, view, and interpret data and to exchange data and messages. Physicians personally answer patients’ questions within 24 hours via system. Frequency of feedback not specified.	Quasi-experimental studies: n = 10 Two conditions: Intervention: n = 5 Control: n = 5 (cross-over design, switch half way through the trial) Both groups comparable concerning intervention time and inclusion criteria (inadequate metabolic control, DM duration greater than 5 years)	a) Decreased HbA_1c_ (I: 8.4 to 7.9, *P* = .053); increased in C (8.10 to 8.15, *P* = .58). NSD between groups. c) Patients transmitted 3524 blood glucose readings, 1649 daily insulin adjustments, 24 exercise reports, and 10 diet modifications. Electronic communication with caregivers was limited; a total of 63 text messages were sent by all patients. Caregivers sent 118 text messages to patients (feedback and therapy modifications). Caregivers performed more therapy changes in I than in C due to the ability to assess patient’s condition on a frequent basis. d) Increased confidence in daily self-management. e) Patients found the system has high utility despite several technical problems.
[24] United States 2002 3 months, follow-up unknown	Primary care. Email for communicating disease management issues between Veterans Affairs primary caregiver and pharmacists. Personal feedback to patients via telephone by pharmacist. Frequency of feedback not specified.	Quasi-experimental studies: n = 65 Two conditions: Intervention: n = 30 Control (usual care): n = 35 Intervention: patients had a recent change made to their therapy to lower blood glucose levels Control: remaining patients of the 65 Comparable HbA_1c_ at baseline in two groups	a) Decreased HbA_1c_ (I: 10.0 to 8.2; *P* < .001; C: 10.2 to 8.6, *P* < .001). NSD between groups. f) Email communication reduced the number of face-to-face and telephone consultations between caregivers. g) Clinical recommendations for altering diabetes care sent via email to primary caregiver resulted in a significant reduction in HbA_1c_ in I.
[25] Spain 2006 12 months, follow-up unknown	Secondary care. Data from glucose meter and vocal messages concerning insulin doses and events are sent via modem (twice a week) to diabetes team (in hospital, members of diabetes team not specified). Diabetes team provides personal feedback. No details provided about form and frequency of feedback.	Experimental studies (RCT): n = 30 Two conditions: Intervention: n = 18 Control (usual care): n = 15 Randomization via random variable generator; baseline data (HbA_1c_, BMI, weight, insulin, DM) and characteristics (age, gender, daily activities) comparable in two groups	a) Decreased HbA_1c_ (I: 8.4 to 7.6; C: 8.9 to 7.6, after 12 months); NSD between groups. b) General health status did not change in groups (SF-12); quality of life improved in I (NS) and C (*P* < .05); significant increase in knowledge in I (*P* < .05) and C (*P* < .05). d) 80% of patients reported that appointments in I did not interfere with daily life; 100% of patients in C reported daily interference with outpatient appointments. f) Time and costs saved by patients. Costs were lower (length of appointment 0.25 h in I versus 0.5 h in C). But 30% of the diabetes team and patient appointments were longer than expected due to technical problems (0.25 h versus 1 h).
[26] South Korea 2006 12 weeks, follow-up unknown	Tertiary care. Clinical data are entered daily in system via website or cellular phone (SMS). Automatic feedback (reminder) is generated in case patient has not forwarded data for more than a week. Personal feedback provided weekly by nurse in tertiary care hospital via SMS, telephone, or Internet.	Observational studies without control group (before-and-after design): n = 42 One condition	a) Patients had a mean decrease of 28.6 mg/dL in fasting plasma glucose (*P*= .006) and 78.4 mg/dL in 2-hour postprandial blood sugar levels (*P*= .003). d) Mean increase in care satisfaction score in I (68.6 to 79.5,*P*= .03).
[27] Italy 2006 56 weeks, follow-up unknown	Care setting not specified. Blood glucose data from a glucose meter are sent via Internet or telephone to the system. Data are automatically analyzed in order to detect metabolic alterations and, if necessary, generate alarms. If necessary, physician (not specified whether GP or specialist in hospital) responds and a message is automatically sent to patient by email or SMS. Frequency of feedback not specified. Based on a general model for the coordination of care (Chronic Care Model).	Experimental studies (RCT): n = 56 Two conditions: Intervention: n = 30 Control (usual care): n = 26 No randomization details; both groups comparable (age, treatment)	a) Location 1 decreased HbA_1c_ (I: 8.52 to 8.30, *P* < .05; C: 8.97 to 8.82). NSD between groups. Location 2 decreased HbA_1c_ (I: 8.40 to 7.75; C: 10.15 to 9.28, after 12 months). NSD between groups. c) Patients transferred 20000 BGL readings and 2000 insulin doses (56 weeks, over 2 locations); the frequency of service usage and quantity of data collected were considered satisfactory. e) Overall usability perception was high (TSQ), especially in adult patients.
[28] United States 2005 At least 24 months, follow-up unknown	Primary care setting. Blood glucose data from glucose meters sent to the diabetes team weekly (team composition not specified) and nurses of the children’s medical care service clinic. Feedback is provided during a clinical, face-to-face session. Online education for school personnel, families, and caregivers is provided on a website. No details provided about form and frequency of feedback.	Observational studies without control group (case series): n = 74 Four conditions: Patients: n = 44 Caregivers: n = 6 Case managers: n = 6 School nurses: n = 18	c) Improved patient-caregiver communication for patients in a remote area. Use of website by nurses increased substantially when it was approved for 3 contact hours of continuing education. d) 40% of patients completed educational modules on the website. e) Users (patients, family, and school nurses) expressed satisfaction with the technology. g) Compliance with school health plans improved compared with baseline.
[29] Australia 2002 6 weeks, no follow-up	Care setting not specified. Internet-based diabetes management systems (myDiabetes, LifeMasters) for evaluation of diabetes management. No details about form and frequency of feedback. Based on the push-pull model for retrieving and seeking information.	Expert opinion based on consensus: n = 5 (1 caregiver, 3 diabetic patients, 1 expert) One condition	e) LifeMasters appeared successful in integrating the health care provider in diabetes management; myDiabetes is effective in providing a communication channel for community creation. LifeMasters appeared a more complete system than myDiabetes (monitoring, personalization, communication, information, technology).
[30] United States 3 months, follow-up unknown	Primary care. Internet-based, diabetes self-management and peer support intervention (chat room). The Diabetes Network was designed to complement medical treatment by providing personalized lifestyle interventions and social support via an Internet-based program accessible from patients’ home. Simplified computers and training were used. Intervention included online blood glucose tracking, twice weekly patient-physician (primary care provider) contact (questions), and message postings on forum (real time chat discussion). Personal dietary advice by primary care provider via website, forum.	Experimental studies (RCT): n = 133 Four conditions: Information only group: n = 33 Peer support group: n = 30 Personal self-management coach condition: n = 37 Combined condition of the three above: n = 33 Randomization by presence or absence of each of the components (peer support, personalized self-management); groups comparable (gender, education, age, years diagnosed)	a) Decreased HbA_1c_ in PSMCC (I: 7.75 to 7.73), in PSC (I: 7.64 to 7.59), in CC (I: 7.46 to 7.28). HbA_1c_ increased in C (7.20 to 7.37 after 3 months) Overall improvement in dietary behavior (reduction of fat intake, improved dietary practices) in 4 conditions, but no significant between-condition differences. b) Slight improvements in quality of life (psychological well-being SF-12) in 4 conditions, especially for PSMCC and CC. c) Two support conditions (PSC, CC) generated significantly more log-ons (M = 61 and 70, respectively, for PSC and CC; M = 40 (PSMCC); M = 25 in IOC; *P* < .02).
[31] United States 2005 12 months, follow-up unknown	Primary care. Clinical data from glucose meters are sent three times a week via Internet to a website. Web-based care management group received a notebook, glucose and blood pressure monitoring devices, and access to a care management website. The site provides educational modules, accepting uploads from monitoring devices and an internal messaging system for patients to communicate with the care manager. Automatic feedback is provided if patients have not forwarded data in 2 weeks. Care manager contacts patients by phone; diabetes nurse communicates with patients about education using the internal messaging system. The care manager responded to queries within 1 working day during office hours.	Experimental studies (RCT): n = 104 Two conditions: Intervention: n = 52 Control (usual care): n = 52 No randomization details; both groups comparable (age, gender, education, metabolic values)	a) Significant decrease in HbA_1c_ in I and C (*P* < .001). A greater decline over time (12 months) in I (10.0, −1.6%) and C (9.9, −1.2%, *P* < .05)_:_ Individuals who persisted with website usage (at least one website log-in every 3 months, *P* < .05) had a greater improvement in HbA_1c_ than usual care. HDL cholesterol rose and triglycerides fell in the Web-based group (*P* < .05). d) Regular data uploads (*P* < .02) were more likely to achieve and maintain reductions in HbA_1c._
[32] United States 2004 3 months, follow-up unknown	Primary care. Web-based disease management program based on an interactive electronic medical record and secure email system. System contains My Upload Meter to automatically upload clinical data daily sent and Diabetes Daily Diary educational website. Automatically generated clinical reminder, email response every weekday (by nurse practitioner in primary care internal medicine clinic). Based on a general model for the coordination of care such as the Chronic Care Model.	Observational studies without control group (before-and-after design): n = 9 One condition	c) If expectations were not met, participants felt their concerns were less valued, and they felt more isolated from their caregiver. d) Participants felt safer having real-time access to their personal health information. They felt more able to manage diabetes by means of seeing laboratory results in the live record at home. e) Frustration with unmet expectations when program did not work as expected (technical failures).
[33] Netherlands 2001 duration not specified, follow-up unknown	Primary/secondary care. Shared-care project whereby all examinations, which take place every 3 months and are performed by the GP, follow standardized procedures. Results are emailed to the diabetologist and laboratory results are automatically sent to both GP and diabetologist. Feedback by post mail from diabetologist to GP.	Observational studies without control group (case series): n = 594 Three conditions: Patients treated in project: n = 336 Patients treated by GP: n = 225 Patients treated in outpatient clinic: n = 33	a) Decreased HbA_1c_ in UDP (7.8 to 6.8, *P* < .0001); mean inclusion duration 3.2 years. Lipid profiles improved in I: plasma cholesterol decreased (6.1 to 5.9, *P* < .0001), plasma triglyceride decreased (1.9 to 1.7, *P* < .0001), and diastolic blood pressure decreased (86 to 83, *P* < .001). d) Data records of UDP cohort were most complete compared to other groups. e) GPs intended to continue participating in UDP despite shared care taking more time. g) Standardized data transfer (protocol driven) between GP, diabetologist, and laboratory established an effective infrastructure for shared diabetes care.
[34] China 2001 12 weeks, follow-up unknown	Secondary care. Dietary and clinical data are recorded in hand-held computer and sent twice a week via a modem to the diabetes team of a hospital diabetes clinic (composition of diabetes team not specified). System generates automatic feedback about content of food.	Quasi-experimental studies: n = 19 Two conditions: Intervention: n = 10 Control: n = 9 Each group used the DMS for 3 months; served as the control group for another 3 months (cross-over design); comparable groups	a) Decreased HbA_1c_ (I: 8.56 to 7.55 after treatment, to 7.84 at end of 12-week project; C: 8.81 to 8.76 after treatment, to 8.40 after end of 12-week project). Mean difference was 0.825 (*P* < .019, n = 19). e) The DMS was acceptable; 95% found it easy to use, and 63% found it useful.

^*^ a) clinical values, b) quality of life, c) interaction, d) self-care, e) usability of technology, f) cost reduction, g) transparency of guidelines, h) equity (availability of health care to everyone) BG, blood glucose; BGL, blood glucose levels; BMI, body mass index; C, control group; CC, combined condition; DM, diabetes mellitus; DMS, diabetes monitoring system; GP, general practitioner; I, intervention; IOC, information only condition; M, mean; NS, not statistically significant; NSD; not statistically significant difference, PSC, peer support condition; PSMCC, personalized self-management coach condition; SBGM, self blood glucose monitoring; SMS, Short Message Service; TSQ, Telemedicine Satisfaction Questionnaire; UDP, Utrecht Diabetes Project; WHO, World Health Organization

**Table 4:** Overview of videoconferencing and combined interventions

Reference, Country, Year, and Duration of Intervention	Care Setting and Intervention	Study Design, Inclusion Criteria, and Data Gathering Methods	Reported Findings (Improvements)
[35] Austria 2002 12 months, follow-up unknown	Primary/secondary care. Patient and GP consult specialist in diabetes center via PC fitted with videoconferencing card connected to a single ISDN line. Personal feedback (diabetologist) on therapy change during video session. Based on organizational learning theory.	Observational studies without control group (before-and-after study): n = 154 One condition	a) Decreased HbA_1c_ (8.1 to 7.8,*P* < .05, after 12 months); systolic blood pressure (156.0 to 148.0 mmHg, *P* < .0005); diastolic blood pressure (88.0 to 83.0 mmHg, *P* < .0005). GPs measured late complications and metabolic parameters more frequently during the project than they did before. e) Technical quality of therapeutic counseling via videoconferencing was sufficient, good enough to evaluate the clinical course of foot ulcer; duration of interview via videoconferencing with patients was on average 12 min (range 4-23 min). f) Reduction of hospital admissions from 12 before I to 7 (during 1 year); duration of hospitalization for whole patient group for treatment of acute complications was reduced (110 to 68 days per year).
[36] China 2005 8 weeks, no follow-up	Secondary care. Patients in a community center and specialist in hospital connected via a large-screen television in the center and a digital camera for better visualization of skin and wound condition and Internet protocol networking videoconferencing units with televisions. Educational sessions (caregiver not specified) regarding diet and ideal body weight, foot care, glucose monitoring, and exercise prescription. No details provided about form and frequency of feedback. Based on a model of service delivery using the group setting for education regarding disease management of elderly people with diabetes.	Observational studies without control group (before-and-after study): n = 22 One condition	a) Reduction in total calorie intake (energy:*P*= .000; carbohydrates:*P* < .002; protein:*P* < .039; fat:*P* < .001) and BMI (*P* < .005). b) Improvement in disease-specific and generic measures of quality of life (SF-36, physical:*P*= .000; general health:*P* < .001; vitality:*P* < .005; social:*P* < .013; emotional:*P* < .019; DQOL:*P*= .000). d) Improved disease knowledge (mean score 7.91 to 13.05); better diabetes control (measured by 2-hr hemastix). c/e) Patients accepted videoconferencing, preferred face-to-face interaction; staff found system easy to use.
[37] Norway 2005 duration not specified, follow-up unknown	Secondary care. Treatment of diabetic foot ulcers whereby a nurse goes to the patient’s house with videophone and laptop and consults physician (specialist in hospital). An online ulcer record system is available capable of notifying the physician by SMS messages. Feedback from physician via videophone.	Observational studies without control group (case series): n = 20 Two conditions: Workshops: n = 15 Pilot test: n = 5	e) Staff and patients found equipment easy to use. f) Patients saved time (no travel to hospital, no waiting time). g) Shared documentation enhanced treatment (coordination).
[38] United States 2001 24 months, follow-up unknown	Primary care. Video visits in addition to skilled nursing visits (Visiting Nurse Association). Patient station in the home has camera with close-up lens. Patient and clinical station linked together over ordinary telephone lines via standard modem. No details provided about form and frequency of feedback.	Experimental studies (RCT): n = 171 Two conditions: Intervention (1 video visit, 1 home visit, 1 video visit): n = 86 Control (skilled nursing home visits only): n = 85 No randomization details; groups comparable (gender, average age, mean diabetes severity score, mean number of comorbidities)	f) Cost savings without compromising quality; videoconferencing has the potential to provide the same number of patient encounters at lower costs; financial benefit increases as the duration of the patient care episode increases. Fewer videoconferencing patients required recertification after 60 days in I compared to C (23% versus 25.6%, *P* < .001); 63.7% of videoconferencing patients were discharged to home care compared to 39% of C group (*P* < .001). 28% of C was hospitalized during 60 days compared to 10% of videoconferencing patients (*P* < .05).
[39] United States 2003 3 months, in case of success, follow-up	Secondary care. Patients report blood sugar levels, injections, and food intake daily either by telephone, videophone (analogue videophone connected to a television), or email. Psychological staff provides advice on changing and maintaining behavior (phone, videophone, or email). Diabetes nurse ensures medical needs.	Observational studies without control group (case series): n = 5 Three conditions: Telephone intervention: n = 3 Videophone intervention: n = 1 Email intervention: n = 1	a) Decrease of HbA_1c_ (for each child: from 9.7 to 8.5; from 8.7 to 7.1; from 13 to 6.1, from 10.2 to 9.4; from 10.9 to 7.9, after 3 months). d) Better self-control (managing the sending of blood sugar); no hospitalizations; no school absences.
[40] Canada 2002 3 months, follow-up unknown	Secondary care. Video visits whereby patient at home communicates with physician in hospital. Equipment not specified. No details provided about form and frequency of feedback. Based on Donabedian’s principle of quality of care.	Observational studies without control group (case series): n = 25 Four conditions: Patient group: n = 8 Nurse group: n = 13 Physicians: n = 7 Managers: n = 7	e) Patients and managers identified a higher degree of readiness for videoconferencing in patients because of the potential to support independence in their homes and in managers because of efficiency of the system. Patients wanted to maintain their level of health but with minimum intrusiveness; caregivers were more interested in measurable clinical outcomes (blood pressure, glucose); managers focused on cost-effectiveness.
[41] United States 2003 3 months, follow-up unknown	Primary care. Patients at remote telemedicine site connected to nurse educator and dietitian in diabetes center through videoconferencing. Both sites equipped with PC, digital camera, and a conference system. Three one-on-one monthly educational sessions (nurse and educator). Feedback given by nurse educator and dietitian during sessions.	Experimental studies (RCT): n = 46 Two conditions: Intervention: n = 24 Control (education in person): n = 22 Randomization via random permuted blocks; groups comparable (age, gender, BMI, duration of diabetes)	a) Decrease in HbA_1c_ (I: 8.7 to 7.8,*P* < .001; C: 8.6 to 7.6,*P* < .001, after 3 months). NSD between groups. b) Reduced diabetes-related stress was observed in I and C (*P* < .007). NSD between groups. d) More positive appraisal of their diabetes (*P* < .05) in both groups. e) Most patients who received videoconferencing felt comfortable with videoconferencing and found it very convenient; overall satisfaction was high (score 4.3/5). Satisfaction with treatment increased in both groups (*P* < .001). NSD between groups.
[42] United States 2005 at least 24 months, follow-up unknown	Secondary care. Nurse and patient in clinic consult physician in hospital via videoconferencing equipment and hand camera, semi-monthly. An educational website covers the basics of diabetes care. No details provided about form and frequency of feedback.	Observational studies without control group (before-and-after study): n = 44 One condition	e) Over 90% of patients and family members expressed satisfaction with videoconferencing. f) Reduced hospitalizations (before I, on average 13 per year [47 days]; after I, 3.5 per year [5.5 days]). Reduced emergency department visits (from 8 to 2.5 per year). The visit interval decreased from 149 to 89 days as the bi-weekly telemedicine clinics replaced quarterly clinics. h) Improved access to specialized health care via videoconferencing (underserved area), in combination with online education improved health status.
[43] Australia 2003 28 months, follow-up unknown	Secondary care. Video consultation between patient (groups) at regional center and pediatric specialist in hospital used in three ways: (1) routine specialist clinics via videoconference using PC with videoconferencing equipment, digital camera, and ISDN line, (2) ad hoc patient consultations at time of urgent clinical need, and (3) education to staff and patients throughout the state of Queensland. Personal feedback by specialist in hospital to patient and staff during video session. Frequency of feedback not specified.	Observational studies without control group (case series): n = 170 Three conditions: Routine consultation: n = 135 Complication consultations with 1 patient: n = 25 Education sessions: n = 10	f) Reduced travel time for specialist hospital staff (by conducting clinics via videoconferencing), while maintaining patient contact. h) Improved access to specialist services (telepediatric) from rural and remote areas.
[44] United States 2000 3 months, follow-up unknown	Primary care. Monitoring metabolic values and dietary behavior from patients’ home unit to primary care clinic, family practice, or internal medicine at the medical center. Weekly patient-nurse consultation through videophone over a telephone line to discuss metabolic values and dietary behavior. Email contact maintained between case manager, specialist, and the family practitioner. Nurse case manager provides advice once a week during session; primary care physician is contacted once a month (for advice).	Experimental studies (RCT): n = 28 Two conditions: Intervention: n = 15 Control (no information available about control group): n = 13 Randomization (stratified based on age, gender, microalbumin, creatinine, HbA_1c_) Comparable groups	a) Decreased HbA_1c_ (I: 9.5 to 8.2,*P* < .05; C: 9.5 to 8.6, after 3 months). NSD between groups. Mean weight reduction (4%).
[45] United States 2004 12 months, follow-up unknown	Secondary care. Self-management therapy video consultation (on nutrition) between patient at home and specialist in hospital. The telediabetes program had been in operation for 10 years. Equipment used not specified. Registered nurse conducts educational session with patient by videoconferencing.	Observational studies without control group (case series): n = 60 One condition	c) Sustainability of the telediabetes program depends on a feedback system; the effectiveness of the process depends on an interactive ongoing collaboration between patient and caregiver. f) Reduced travel time for patients and caregivers. g) Administration took a long-term view of the value of telemedicine service; service delivery followed national diabetes standards and a well-defined cycle of care within a long-term quality improvement program and consistent education program resulted in sustainable diabetes care. h) The system provided access to specialized health care in remote areas.
[46] United States 2004 12 weeks, follow-up unknown	Secondary care. Physician and physical therapist in hospital connected with patient and nurse in medical center for the treatment of diabetic foot ulcers. Both equipped with a videoconferencing unit and a television monitor. The hospital has a hand-held camera for real-time transmission of close-up images of the foot and a document camera for real-time transmission of foot x-ray images. Personal feedback by nurse during weekly session.	Quasi-experimental studies: n = 140 Two conditions: Intervention: n = 20 Control (face-to-face foot program): n = 12 Comparable groups (age, wound condition)	a) No difference in I and C in the average forefoot ulcer healing time, the percentage of ulcers healed in 12 weeks, or the adjusted healing time ratio. e) Equipment appeared easy to use and provided clear viewing of foot lesions and x-rays. Patients appeared well satisfied with use of technology. f) Patients saved travel time. h) Patients appreciated the convenience of being treated at their local facility (had more access to specialized care).
[47] China 2002 18 weeks, follow-up unknown	Primary care. Education in small patient groups in health center given by nurse in diabetic center through videoconferencing equipment connected by a local area network. Four sessions, each lasting 2 hours. Personal feedback (from diabetes nurse) during sessions.	Observational studies without control group (case series): n = 41 One condition	c) Videoconferencing enabled community nurses in primary care to link with nurse specialist in diabetes center to provide diabetes education in small groups. d) Diabetes education conducted via videoconferencing was highly acceptable (mean total score [TSQ] was 61.9/75). e) Significant positive correlation between age and satisfaction (r = 0.39); the older the patient, the higher the level of satisfaction with videoconferencing and with caregiver; no relationship between satisfaction with videoconferencing and baseline HbA_1c_. Lack of a perceived need to have assistance while using equipment and the perceived ability of videoconferencing to meet health care needs were most important predictors of satisfaction (accounted for 82% of the variance in satisfaction). f) Patients saved travel time and waiting time.
[48] United States 2005 24 months, follow-up unknown	Primary care. Intervention consists of three parts: 1. Hand-held in-home messaging device (Health Buddy) with disease management dialogues: Patients answer a daily series of questions and the care coordination staff of Veterans Health Administration (staff composition not specified) review responses daily to determine the level of risk for health care emergencies. 2. Telemonitoring with two-way audio-video connectivity that allowed for weekly monitoring of glucose and vital signs. 3. Videophone with two-way audio-video connectivity, not including biometric monitoring. Patients followed up on a weekly basis for biometric info. Care coordinator reviews data daily to determine risks. Based on Wagner’s Chronic Care Model.	Observational studies without control group (before-and-after study): n = 445 One condition	b) Significant improvement in health-related quality of life during 1 year (role-physical functioning: *P* = .02; bodily pain: *P* = .005; social functioning [SF-36]: *P* < .05). e) Patients found equipment easy to use (> 95% of patients). f) Reduction in proportion of patients who were hospitalized (50% reduction, *P* < .0001), in emergency room visits (11% reduction, *P* < .04), in average number of bed days of care (decreased an average of 3.0 days, *P* < .0001). Patients were 35% more likely to have had one or more need-based primary care clinic visits (*P* = .0004).
[49] United States 2005 24 months, follow-up unknown	Primary care. See [48]. Comparison of weekly monitoring with care coordinator versus daily monitoring with home message system. Personal feedback if necessary: caregiver calls patient or facilitates an appointment. Instant camera with grid film for following diabetic wounds for aggressive wound management (weekly monitored group targeted patients with active diabetic wounds). Patient takes two pictures of wounds and mails them to care coordinator. Care coordinator reviews data daily to determine risks. The daily monitored group consists of diabetics who had wounds that required careful monitoring.	Quasi-experimental studies: n = 297 Two conditions (two different monitoring intensities): Weekly monitoring, intensively monitored: n = 197 Daily monitoring, less intensively: n = 100 In weekly monitored group, patients were younger; in daily program, more patients were married; both groups comparable in clinical and sociodemographic characteristics	a) Decreased HbA_1c_ in I (weekly monitoring, from 8.3 to 8.1,*P*= .22) and in C (daily monitoring, from 8.7 to 8.8,*P*= .78) after 24 months. NSD between groups. Adjusted mean values HbA_1c_ in I (weekly monitoring) from 8.1 to 7.8 (*P*= .20) and in C (daily monitoring) from 8.6 to 8.7 (*P*= .79) after 24 months. NSD between groups. f) Proportion of one or more hospital admissions decreased in daily monitoring group (77 to 43, *P* < .01) and increased in the weekly monitoring group (73 to 106, *P* < .01). The change in the average number of hospital bed days was 8 days lower in daily monitored group than in the weekly monitored group (*P* < .0001). Unscheduled primary care clinic visits were lower in the daily monitoring group (67 to 16) than in the weekly monitoring group (108 to 116); significant difference between the two groups (*P* < .01).
[50] United States 2005 12 months, follow-up unknown	Primary care. Patient-centered care coordination / home telehealth program based on Wagner’s Chronic Care Model provides self-management and decision support via electronic reminders and care coordinator. The system used an in-home dialogue box (via patients’ cell phone) to answer questions about health status. Answers were sent daily over the Internet to care coordinator who responded in case of alarming values. A two-way audio video connectivity and videophone were also used (see [48]).	Controlled observational studies (cohort studies): n = 800 Two conditions: Intervention: n = 400 Control (no intervention): n = 400 Propensity scores were used to improve the match between I and C. A difference-in-differences approach used to measure the effects of the intervention on service use	f) Significant difference between I and C in need-based primary care visits, increasing in I (7.6%) and decreasing in C (12%) (*P* < .01). The likelihood of 1 or more emergency department visits decreased in I and C (significant differences between groups, *P* < .0001). I group had a lower relative likelihood of having 1 or more hospitalizations than patients in the control group (control for HbA_1c_, NS difference between I and C). h) Increase in access to care in I.
[51] United States 2005 5 months, follow-up unknown	Primary care. Monitoring blood glucose via a home telemedicine unit coupled with care management delivered from the diabetes centers at two hospital-based hubs. Patients can upload monitoring data, send secure email, access an educational website, and use two-way video / voice conferencing. No details provided about form and frequency of feedback.	Observational studies without control group (case series): n = 5 One condition	e) Technology-related problems (telecommunication, connectivity) were the primary cause of installation difficulties (in patients’ homes). Patient education and training are the most critical success factors. Patient education and training accounted for two thirds of the in-home time for installation of equipment. Nurse installers are patient-centric rather than technology-centric.

^*^ a) clinical values, b) quality of life, c) interaction, d) self-care, e) usability of technology, f) cost reduction, g) transparency of guidelines, h) equity (availability of health care to everyone) BMI, body mass index; C, control group; DM, diabetes mellitus; I, intervention; NS, statistically not significant; NSD, not statistically significant difference; TSQ, Telemedicine Satisfaction Questionnaire

### Studies on the Effects of Teleconsultation

#### Settings and Interventions

Interventions took place in secondary care settings [13,15,17,19,22,23,25,34] and in primary care [24,28,30-32] (see Table 3). To improve the reliability of monitoring, clinical data such as HbA_1c_ and insulin dose were usually sent and analyzed automatically (18/22 studies). In most settings, glucose meters, palmtops, and/or cell phones were used to send data (n = 15). To enhance disease control, feedback was given via computer-generated reminders whenever values were alarming [13,14,20,21,26,27,32]. In some cases, caregivers provided personal feedback to instruct patients in case of alarming values [15,17,22,24,25,30,33,34].

Inclusion of patients in the intervention groups included such criteria as being diagnosed with type 1 [13,15,18,19,22,23,25,28] or type 2 diabetes [24,26,30,32,33], being compliant with therapy [13,22,24], being motivated to take part in the intervention [14,15] having a caregiver taking part in the intervention [17,24,31,33,34], living in the region [17,21,30,32], demographics such as being younger than 30 years [19,22] and being economically disadvantaged [17,22,28,30,32], having insulin problems [15,19,25], and poor metabolic control (HbA_1c_> 8%) [19,22-25,31]. As well, certain conditions needed to be met, such as being able to handle the technique [15,17,18,20,26,30], having followed a structured diabetes education program [15], and having access to the Internet [14,20,26] or a (cell) phone [14,17,20,21,26,30].

Though teleconsultation is generally assumed as the solution for better disease management and care coordination of diabetic patients, the preference for this kind of technology compared to other options has not been clearly stated. Teleconsultation is supposed to be cost-effective, to deliver continuous care, and to foster time-efficient communication between patients and caregivers [13,14,16,17,19,21,23,31]. Most teleconsultation interventions (n = 18) were aimed at improving clinical values, investigating usability of technology (n = 15), intensifying interaction by means of information exchange, either among caregivers or between patient and caregivers (n = 14), and enhancing self-care (n = 12).

#### Effects of Teleconsultation at Clinical Level

HbA_1c_ levels were measured in eight RCTs [14,15,19,22,25,27,30,31], but only six were suitable for meta-analysis. One trial studied only children [22] and was therefore not included in the meta-analyses. Another trial [14] reported that the variance of HbA_1c_ values was significantly lower in the experimental group compared to the control group. This study was excluded because using an imputation technique was unadvisable as data provided by the author differed from the published data. Changes in HbA_1c_ values were calculated from baseline and follow-up means and standard deviations. The Jadad quality score of the trials was either 2 or 3 (Table 5).

**Table 5:** Randomized controlled trials with HbA_1c_ data (see Multimedia Appendix for full tables containing inclusion criteria and data gathering methods)

Study	Trial Duration (months)	Intervention			Control			Quality Score [12]
		N	Baseline and Follow-Up Values ± SD	Mean Difference ± SD	N	Baseline and Follow-Up Values ± SD	Mean Difference ± SD	
Biermann [15]	4	27	8.3 ± 2.3 6.9 ± 1.3	−1.4 ± 2.0^*^	16	8.0 ± 2.1 7.0 ± 1.0	−1.0 ± 1.82	3
McKay (a) [30]^†^	3	37	7.75 ± 1.33 7.73 ± 1.42	−0.02 ± 1.38^*^	33	7.20 ± 1.36 7.37 ± 1.49	0.17 ± 1.43	3
McKay (b) [30]^†^	3	30	7.64 ± 1.71 7.59 ± 1.66	−0.06 ± 1.69^*^	33	7.20 ± 1.36 7.37 ± 1.49	0.17 ± 1.43	3
McKay (c) [30]^†^	3	33	7.46 ± 1.35 7.28 ± 1.28	−0.18 ± 1.32^*^	33	7.20 ± 1.36 7.37 ± 1.49	0.17 ± 1.43	3
Farmer [19]	9	47	9.2 ± 1.1 8.6 ± 1.4	−0.62 ± 2.42^‡^	46	9.3 ± 1.5^§^ 8.9 ± 1.4	−0.38 ± 2.36	2
McMahon [31]^||^	12	52	10.0 ± 0.8	−1.6 ± 1.4	52	9.9 ± 0.8	−1.2 ± 1.4	2
Jansa [25]	12	18	8.4 ± 1.2 7.6 ± 0.9	−0.8 ± 0.74^§^	15	8.9 ± 1.3 7.6 ± 0.7	−1.3 ± 0.64	3
Larizza [27]^¶^	12	15	8.40 ± 2.53 7.75 ± 1.16	−0.65 ± 2.20^*^	14	10.15 ± 3.25 9.28 ± 2.34	−0.87 ± 2.90	2

^*^Imputation technique.

^†^Intervention (a) was personal self-management coach (n = 37), intervention (b) was peer support (n = 30), and intervention (c) was combined condition (n = 33).

^‡^Calculated using 95% CI.

^§^Calculated using *P* values provided by author.

^||^Precise follow-up data were not reported.

^¶^Data from location 2 (adults) of the study, see Table 3.

**Figure 2.**
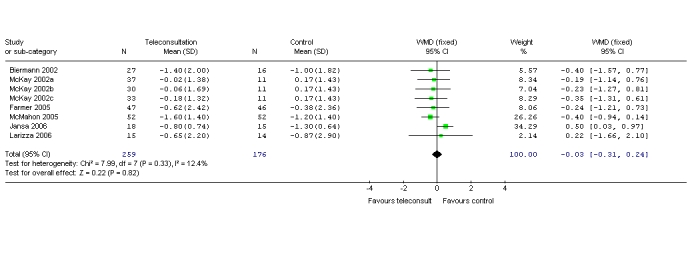
Comparison of changes in HbA_1c_ control versus intervention

Table 5 presents the mean difference between the baseline and follow-up HbA_1c_ values. These values were either reported in the paper or provided by the authors. None of the interventions were blinded since blinding of participants with respect to study status is almost impossible in clinical trials of behavioral interventions. The method of randomization was not clear in two of the six RCTs; a description of withdrawals and dropouts was given in five studies. The pooled reduction in HbA_1c_ was not statistically significant (weighted mean difference [WMD] 0.03; 95% CI = −0.31 to 0.24). Figure 2 shows the mean difference and WMD of the mean difference between baseline and follow-up HbA_1c_ values. There was no significant statistical heterogeneity among the pooled RCTs (*χ* ^2^_7_ = 7.99, *P* = .33). The pooled RCTs included patients with type 1 diabetes [13,15,19,25], type 2 diabetes [31], and unspecified diabetes [27,31]. Glucose monitoring took place via a telephone network [14,15,19,25,27], the Internet [14,30,31] in primary care [30,31], secondary care [15,19,25], or integrated care [14] settings, or the care setting was not specified [27]. None of the pooled RCTs showed a significant difference in HbA_1c_ between intervention and control groups. The trials varied in duration from 3 to 12 months.

Two RCTs reported a decrease in HbA_1c_ values. In one study [14] (DM type 1, 2, or unspecified), both the intervention and control groups had significantly different variances after 6 months (F test, *P* < .05), confirmed by the results of the randomized subset of patients (*t* test, *P* < .05) (see Table 3, reported findings under a). The other study [22] analyzed the HbA_1c_ values of children (DM type 1) (see Table 3, reported findings under a). There was no significant difference between the two groups and no significant within-group difference between initiation and completion of the study (6 months). Some observational or quasi-experimental studies showed improved metabolic control with respect to HbA_1c_ [13,16,23,24,33,34], diabetes regulation [33], and glucose, lipid profiles, and blood pressure [31,33]. The improvements were not significant compared to the control group (usual care). The improvements with regard to metabolic control were achieved by means of Web-based care management programs providing patients (mostly DM type 1) who have poor metabolic control with automatic data transmission, educational modules, and messaging systems for communication and personal feedback.

Quality of life improved in three studies [17,25,30] (see Table 3, reported findings under b). These studies measured different aspects of quality of life. In one study [17], a mean improvement was realized in the mental and physical status of patients after 6 months of intervention. In the second study [25], an improvement was observed in quality of life (DQOL) and in knowledge (DQK2) in the intervention and control group (usual care). In the third study [30], a slight improvement was reported in psychological well-being, especially in the personal self-management and combined condition.

#### Effects of Teleconsultation at Behavioral Level

Ten studies [13,16,17,18,22,23,27,28,30,32] reported improvements in patient-caregiver interactions (see Table 3, reported findings under c) with respect to a higher frequency of information exchange (about treatment) and increased metabolic data transmission [16,17,23,30]. In study 16, the intervention was compared to usual care, while in study 17 and 23, the effects were demonstrated in the intervention phase of a cross-over design. In study 30, two of the four care conditions (personal self-management and personal self-management combined with peer support) showed a significant improvement in patient-caregiver interaction. A communication network improved the availability and completeness of data among caregivers [16,23]. Intensity of contacts increased [17,23,30] via daily monitoring and automatic feedback when values were alarming [17], via personal feedback to patients’ requests for advice [23] or via an Internet-based program for self-management and social support [30].

Improved self-care was observed in 10 studies [17,18,20,23,25,26,28,31,32,33] (see Table 3, reported findings under d). Patients checked their blood glucose more often [18,31], experienced a better understanding of their medical condition [17,26], and were better able to manage their disease after using the technology [17,18,20,23,25,28,32,33]. Better self-care was achieved in interventions with personal feedback [17,18,20,23,25,26,28] and/or education [28,31,32]. In one study [31], the improvement was significant compared to usual care. Regular data uploads were more likely to achieve and maintain reductions in patients’ HbA_1c_.

#### Effects of Teleconsultation at Care Coordination Level

In general, most patients were satisfied with the technology (see Table 3, reported findings under e). The technology (eg, glucose meters, hand-held electronic diary) was acceptable for patients [13,23,28,29], was reliable and helpful for caregivers [13,28,33], and appeared easy to use [15,17,21,27,34]. Technical problems and unfulfilled expectations frustrated patients [32]. The availability of metabolic data and the possibility of consulting a caregiver within minimal time and without traveling enhanced patients’ feeling of security [15].

Adoption of the technology was demonstrated by a significant increased proportion of transmitted blood glucose data in the intervention group [19]. In most cases, patients were trained to master the equipment [15,17,18,19,20,22,23,25,26,28,30]. Two studies [18,23] reported on the implications of implementing technology in diabetes care. Web-based management of diabetes care [18] changed tasks and duties of the diabetes team as the system required extra competency of nurses in insulin dose adjustments. Electronic communication and frequent blood glucose transmission led to more changes in therapy compared to usual care [23].

Five studies [15,17,21,24,25] reported cost reductions (see Table 3, reported findings under f). Cost reductions concerned saving of consultation time compared to usual care [15,25]. However, teleconsultation significantly increased physician’s time since patients tended to call more often [15] and more time was needed to handle technical problems [25]. Costs for technical equipment, telephone, and data transfer were compensated by the cost savings [15]. Costs were calculated as savings per year per patient, reduction of overall utilization and charges after one year, and treatment time required of caregivers. Costs were measured by means of interviews, nonstandardized questionnaires, and by retrieving data from visit logs. A cost analysis was carried out in one study [15] with an estimation of total costs of teleconsultation in an optimized scenario, including a comparison to usual care.

Enhanced transparency was realized in three studies [24,28,33] (see Table 3, reported findings under g). Clinical guidelines for altering diabetes care (a treatment algorithm for metabolic control) resulted in a reduction in HbA_1c_ in patients with poorly controlled diabetes; however, the difference in reduction between intervention and control groups (usual care, actively managed comparator group) was not significant [24]. Protocol-driven data transfer between caregiver and laboratory provided more complete patient records and a significant decrease in metabolic values (HbA_1c_, lipid profiles, and blood pressure) [33]. Online education of school personal enhanced compliance with school health plans [28].

### Studies on the Effects of Videoconferencing and Combined Interventions

#### Settings and Interventions

Interventions took place in secondary care settings [36,37,40,42,43,45,46] and in primary care [35,38,39,41,44,47,48,49,50,51]. Combined interventions [48-51] were all used in primary care settings, often in underserved or remote areas, to allow videoconferencing to supplement teleconsultation by enabling direct interaction between patient and caregiver(s) [49,50]. Videoconferencing involved real-time contact between the patient at home [37,38,39,40,44,45] or in a local clinic or center [35,36,41,42,43,46,47] and a caregiver in the hospital or diabetes center via video equipment. In 11 cases, patients had contact with one caregiver, while in six cases a team of various caregivers interacted with patients. In studies aimed at patient education [36,41,43,47], consultation took place between patient groups and caregiver(s). Videoconferencing was used for ulcer treatment [35,37,46], for patients discharged from hospital but still needing care [38], for injections and blood sugar control [43],and for general diabetes management [40,42,45]. Feedback was provided mostly during the video sessions or in combined interventions by telephone [49,50] or email [51].

Inclusion criteria for the videoconferencing and combined interventions (see Table 4) included being diagnosed with DM type 2 [35,36,44,47], age [36,39,41,44,47,48,49,50], being treated longer than 1 year [35], being (frequently) referred to a diabetes specialist [47], having a complex medical condition [39,48,49,50], poor metabolic control [39], being at high risk of expensive service visits [48,49,50], being a pediatric patient [42,43], having limited mobility [36], having a caregiver taking part in the intervention [38,39,40,41,45], not having had diabetes education for at least 1 year [41], and having phone access [50]. Three studies did not mention inclusion criteria [37,46,51].

Videoconferencing was chosen because it permitted experts from the hospital to be present in the patient’s home while maintaining the continuity and quality of treatment to support disabled or underserved patients [39-41,43,46,49,50]. Combining the two modalities was motivated by the fact that videoconferencing supplements teleconsultation by enabling direct interaction. It was expected that through videoconferencing it would be possible to explain the effects of teleconsultation more accurately [48]. Videoconferencing (and combined) interventions were mainly aimed at cost reduction (n = 14), clinical improvement (n = 11), usability of technology (n = 8), self-care (n = 8), and quality of life (n = 7).

#### Effects of Videoconferencing and Combined Interventions at Clinical Level

Improved metabolic control was observed in six studies [35,36,39,41,44,49] (see Table 4, reported findings under a).

HbA_1c_ decreased [35,36,39,41,44,49], systolic and diastolic blood pressure decreased [35], total calorie intake decreased, body mass index and glycemic control improved [36], and low density lipoprotein cholesterol decreased [41]. Due to the fact that data regarding standard deviations could not be retrieved, a meta-analysis could not be conducted on the RCTs reporting HbA_1c_ levels.

HbA_1c_ decreased via therapeutic counseling by videoconferencing [35] and by means of daily monitoring of food intake and blood sugar levels via videophone, email, or phone [39].

A comparable reduction in HbA_1c_ was found as a result of educational sessions by videoconferencing with patients in remote areas compared to in-person education (no statistically significant difference between intervention and control groups) [41] and also by monitoring metabolic values and dietary behavior from patient’s home with videophone and email (no statistically significant difference between intervention and control groups) [44]. In another study, a hand-held in-home messaging device (Health Buddy), a two-way audio-video link, and a videophone were used to compare weekly monitoring (with a care coordinator) to daily monitoring (with the home message system). HbA_1c_ decreased in both groups (no statistically significant difference between groups) [49].

Most interventions were directed at patients with DM type 2, with poor metabolic control, or with complex medication conditions and a high risk of expensive service visits.

Improvement in quality of life was reported in three studies [36,41,48] (see Table 4, reported findings under b). Two studies [36,41] concerned educational interventions. In one, videoconferencing (video and digital camera) took place in a community center with patients who had limited mobility and skin and foot care (wounds) problems [36]. In the other study, patients at a remote site were connected by videoconferencing, digital camera, and personal computer to a diabetes center [41]. One intervention [48] consisted of a home message system that allowed monitoring and communication by videophone. Quality of life improvements were reported for physical functioning [36,48], general health [36], emotional well-being [36], stress reduction [41], and social functioning [36,48]. Only in one study a control group was used, but no significant difference was observed between the intervention (tele-education) and control groups (education in person) [41].

#### Effects of Videoconferencing and Combined Interventions at Behavioral Level

Patient-caregiver interaction improved in three observational studies [36,45,47] (see Table 4, reported findings under c). Patients developed a wider social network, creating bonds with both other patients and with caregivers. An interactive ongoing collaboration between patient and caregiver was found to be important for the effectiveness of self-management therapy [45]. Videoconferencing enabled communication between caregivers, to provide education in small groups [47].

Self-care improved in four studies [36,39,41,47] (see Table 4, reported findings under d). Self-care improved by management of blood sugar transfer [39] and increased knowledge allowing patients to cope with diabetes in a better way, thus improving self-care [36]. Patients developed a more positive view of their diabetes [41]. Education via videoconferencing appeared highly acceptable for patients [47]. The improvements in self-care [36,41,47] took place in care settings that were particularly directed at (group) education.

#### Effects of Videoconferencing and Combined Interventions at Care Coordination Level

Equipment for videoconferencing consisted of a personal computer with video card [35,38,41], equipment with a television [36,42,43,46,47], or a videophone [37,39,44]. A document camera or visualizer was used to show patient records, x-ray images [36,41,43,46], blood sugar values, and pictures of foot ulcers, skin conditions, and wounds. A hand-held camera was used for showing body sites (eg, ulcers) [36,38,42,46,50]. The combined interventions used hand-held in-home messaging devices (Health Buddy) and a videophone for monitoring glucose [48-50].

Videoconferencing equipment [35-37,39-42, 46,47,48,51] appeared convenient and easy to use; caregivers found the photographic images reliable and valid [46] (see Table 4, reported findings under e). seven studies, patients and caregivers were trained to use the equipment [37,38,42,47,49,50,51].

Satisfaction with videoconferencing depended on education and training [51], assistance while using the equipment, and age; the older the patient, the higher the level of satisfaction with videoconferencing [47].

Cost reduction was reported in 11 studies [35,37,38,42,43,45,46,47,48,49,50] (see Table 4, reported findings under f). Cost savings concerned reduced health care utilization [35,38,42,48,49,50], lower treatment costs [38,42], more need-based primary care clinic visits (permitting just-in-time preventive care instead of just-in-case care) [48,49,50], and reduced travel costs for patients [37,46,48] and caregivers [43].

Reduction in health care utilization costs was achieved with respect to hospital admissions [35,49] emergency department visits [42,48,50], hospitalizations [38,42,48], number of bed days of care [48,49], and discharges to home care [38]. Lower treatment costs refer to the potential of videoconferencing to provide the same number of patient encounters at lower cost, decrease patient referrals [38], and replace conventional visits by videoconferencing [42]. Videoconferencing also reduced unscheduled primary care visits [48,49,50]. Studies also associated lower costs with more reliable and valid metabolic control [49,50].

The reductions in costs were found in observational studies without a control group [35,37,42,43,45,47,48]. Costs were calculated during the intervention period and were compared to the costs before the intervention took place (see Table 4, reported findings under f). In three studies [38,49,50], cost reductions were compared to a control group. In one study [38], an economic analysis was carried out on direct and indirect costs occurring at the home health agency level, including labor costs for both the intervention and control groups (skilled nursing home visits only) and costs associated with the implementation of the videoconferencing system. There were no significant differences between intervention and control groups in staff costs (time spent by training, video visits). Total costs per patient per episode were lower for the videoconferencing group, including hospitalization, than for the control group. In two combined interventions [49,50], lower costs were related to more reliable and valid metabolic control. One of these combined studies [49] showed effects of differences in home care monitoring intensities (weekly or daily monitoring) on service costs and clinical outcomes; daily monitoring (transmission via home telehealth technology) significantly reduced the unscheduled primary care clinic visits, the hospital admission rate, and the days of hospitalization. Patients in the daily monitoring group performed better than the weekly (instant camera) monitoring group because of more reliable and valid metabolic control. Although the service cost was reduced, no difference could be found in the clinical outcomes between groups. In the second of these combined studies [50], there were reported differences in health care service use between videoconferencing and conventional care with reference to outpatient services; a difference between intervention and control groups was observed in need-based primary care visits, which increased in the intervention group and decreased in the control group. The likelihood of one or more emergency department visits decreased both in intervention group and control groups, but the intervention group had a lower relative likelihood of having one or more hospitalizations than the control group. Patients who had higher HbA_1c_ levels spent a greater number of days in hospital.

Although videoconferencing saved money, the development and implementation costs (including training of staff) of a new technology are often high, and all kinds of technical problems (and costs) should be taken into consideration. In two studies [38,42], cost savings were compensated by staff training and system costs (including costs of technical deficits). Even when system costs were included, videoconferencing saved money [42] or was estimated to save money on the basis of cost analysis [38].

Enhanced transparency in treatment programs was reported in two studies [37,45] (see Table 4, reported findings under g). Shared documentation via an online ulcer record system enhanced coordination in the treatment of diabetic foot ulcers [37]. A long-term quality improvement program (including national diabetes standards) with an interactive feedback system between patient and caregiver resulted in structured use of staff time [45]. Better access to specialized health care in underserved areas was reported in three studies [42,45,46] and in patients with complex medical conditions in one study [50] (see Table 4, reported findings under e).

### Reported Shortcomings of the Studies

Several publications reported shortcomings concerning disappointing or unexpected study results and problems with implementing the intervention. The most frequently mentioned shortcomings were the lack of a significant difference between the intervention and control groups [14,15,19,24-27,30,33, 35,36,41,46], the inability to measure long-term effects of the intervention [14,17,19,30,44], and the fact that interventions sometimes inherently led to improved results because of a selection bias. Some patient groups benefited more from the intervention than others (eg, patients with poor metabolic control [33], high use of health care [50], motivated patients [22], or inexperienced patients [15,33,48]).

Some publications reported problems with ICT-based care, generally caused by the absence of adequate infrastructure [14,16,27,29,47,50,51] or the logistical difficulties involved in organizing online consultations, with all parties having to agree on a suitable time [50]. Patient-caregiver interaction suffered from the lack of a protocol that could guarantee high-quality communication, leading to information overload [16,17,18,29,33,51]. In some cases, patients considered the technology too complex to master [21,23,30,47,50,51], too time consuming [15,23,30,33], or too costly [21], and some patients were reluctant to cooperate, resulting in unreliable clinical data transmissions [15,18,26,35,47,51]. ICT-based care was thought to reduce the trust and confidential relationship between patients and caregivers [15,18,32,51].

## Discussion

As far as we know, our review is the first to evaluate the benefits of teleconsultation and videoconferencing for diabetes care, in particular with respect to clinical, behavioral, and care coordination aspects. Earlier reviews have focused on usability and costs of technology or considered mainly clinical (glucose and diet) outcomes [2,4,5]. A systematic search and selection process produced only 39 studies. This may appear low, but it is comparable with previous reviews on ICT-based care [3,4,52].

We can conclude that in the period under review (1994-2006), 39 studies had a scope broader than clinical outcomes and involved interventions allowing patient-caregiver interaction. Most of the reported findings concerned satisfaction with technology (26/39 studies), improved metabolic control (21/39), and cost reductions (16/39). Improvements in quality of life (6/39), transparency (5/39), and better access to care (4/39) were hardly observed. In 19 of 39 studies the control group was more or less comparable with the intervention group (see Table 3 and Table 4). It appeared that ICT-based care improved diabetes care compared to usual care; however, the improvements were mostly not statistically significant. In a sense it could be argued that technology did not compromise the care delivery process.

Only a minority of the studies (12/39) considered care settings involving teamwork of various caregivers (eg, nurses, case manager, psychologist, physician, general practitioner), which should be expected in integrated chronic care settings [1,53]. Training was given when implementing the technology, but this was restricted to handling equipment and did not address the technology to solve health care problems, which is a prerequisite for eHealth literacy [54].

The contribution of teleconsultation and videoconferencing to patients’ quality of life and ability to control their disease was not substantial (clinical and statistical), because of a limited intervention period and various shortcomings in research design and in implementing ICT-based care. Although previous reviews have indicated that the impact of technology on behavioral change (interaction and self-care) and on care coordination (cost savings) needs to be clarified to support decisions about the use of technology to supplement care [3,5,52], only limited progress was observed. A possible reason that ICT-based care has not shown a high impact on diabetes care could be the absence of a long-term view on the potential of technology to reduce fragmentation and to improve diabetes care at acceptable costs. In most studies, patients’ perspectives with respect to emotional and social well-being (quality of life) and ability to cope with diabetes are underexposed, just as the feasibility, appropriateness, and meaningfulness of the interventions for care practice are [55]. Moreover, the choice of a specific technology was mostly based on convenience arguments (access to a computer for instance, living in an underserved area) and not related to preferences and specific needs of patients or caregivers to manage diabetes. For example, a study on the attitude toward videoconferencing [40] showed that patients prefer video visits while nurses wanted to deliver hands-on care in patients’ homes. Therefore, it is not certain that the most appropriate technology was used in the most effective way [9], and, consequently, it might be rather premature to say that teleconsultation or videoconferencing as such is the best option to deliver cost-effective and worthwhile services.

Although these shortcomings can be seen as an inevitable part of innovating chronic care, one must consider the benefits of specific technologies to diabetes care to make progress. Based on our review, the benefits of teleconsultation concern the three levels of care. At the clinical level, this implies improvement of metabolic control. Improvements at the behavioral and care coordination level refer to reliable transmission of clinical data (eg, HbA_1c_), intensified patient-caregiver interaction, and enhanced self-care as a result of an improved understanding of the medical condition and higher quality of feedback (quicker response from caregivers and education about self-management).

Teleconsultation interventions [16,17,19,24,25,31] with improvements in clinical, behavioral, and care coordination outcomes can be characterized as Web-based care management programs providing automatic transmission of clinical values, educational modules, and a messaging system for communication and personal feedback (warning messages and instruction). Conditions for implementing the technology were reported in some of these studies, such as using computer-based patient records for electronic data interchange between caregivers; guidelines for writing medical records; a close cooperation between patient, general practitioners, and specialists [16]; access to a care manager to manage diabetes care with technology; and patients who favor ICT-based care [31]. The technology was found not advanced enough to be sufficiently practical and cost-effective [25], and more intensive techniques (like computerized decision support systems) are needed to help patients change their health behavior [19].

Most of the studies reported none or limited information about preference for and persistence of technology for specific patient groups. The observed improvements were based on interventions directed at patients who were able to use the equipment (eg, having experience with cell phones and SMS) [14,17,18,20,21,26], who were well motivated to take part in the intervention [13,14,15,31,33,34], who already had a caregiver taking part in the intervention [17,24], who were economically disadvantaged [17,28], or who had type 1 diabetes that required strict monitoring of blood glucose levels [22,23,25,28]. This might confound the practicability of the results [55,56].

The benefits of videoconferencing can be particularly demonstrated at the usability level (convenient and easy to use) and care coordination level. Videoconferencing appeared to maintain quality of care while producing cost savings in patient at-home care settings. Real-time communication appeared particularly successful in group education, allowing patients to take more proactive roles in managing their diabetes, helping them to feel happier and to develop wider social networks. Monitoring combined with videoconferencing enabled “just-in-time preventive care” instead of more expensive “just-in-case care” and significantly reduced unscheduled clinic visits, hospital admissions, and days spent in hospital. Cost savings should be offset by increased staff costs and the costs of the development and implementation. For instance, increased patient-caregiver interactions or increased need-based primary care may imply an increase in workload. In two [38,42] of the 11 studies on cost savings, the cost reductions were compared to increased system costs.

The results were based on interventions directed at patients in underserved or remote areas, with complex medical conditions (elderly, immobile, or with poor metabolic control), or meeting some practical conditions, such as having access to a physician in the intervention setting, which should be taken into account when implementing videoconferencing in practice, for reasons of selection bias [55,56].

Successful interventions [38,41,48,49] with improvements in clinical, behavioral, or care coordination levels included programs aimed at teaching patients to cope with and control their diabetes, mostly settings in which patients at home consulted with their caregivers at hospitals or diabetes centers via video. Reported conditions for implementing these interventions were training of patients and staff throughout the implementation to learn to deal with the equipment [38], alternative markets to reduce investment costs, like purchasing “used” equipment at reduced costs [38], and a health care system that has an ongoing and well supported clinical infrastructure to support professionals competent to deal with ICT-based care [48].

The observed benefits are consistent with prior reviews regarding cost savings, efficacy of applications, and improved communication between primary and secondary health care providers [4,5,9,52,57]. The scope of the reviews differs from our study, which is particularly aimed at diabetes care.

### Some Limitations of Our Study

Due to the diversity and variance in study designs, inclusion criteria, and a lack of required data, a meta-analysis could not be conducted on the RCTs reporting HbA_1c_ levels (videoconferencing) and other outcomes (quality of life, behavior, and care coordination). In particular, studies on quality of life, behavior, and care coordination used different outcome measures or calculated the same outcome (eg, well-being) in different ways. Lack of required data hampered a statistical combination and therefore may have biased the review’s results. To avoid spurious preciseness, we did not combine observational studies for a meta-analysis.

To evaluate the contribution of technology to diabetes care, we developed a checklist based on principles for chronic care [1,53,58] because existing evaluation systems are directed at usability and acceptability of equipment rather than care service delivery [9]. Future research should validate this checklist. We reported the outcomes of the interventions per level of care, although they are interdependent in a chronic care setting; the usability of the equipment influences the reliability of monitoring and patient-caregiver interaction, which can influence behavior and care coordination [1].

We chose to review various systems of teleconsultation and videoconferencing to shed light on different functions of the systems (monitoring, information exchange, communication) to support diabetes care. This might increase the heterogeneity in our study results.

### Future Research

When patient self-care and care coordination are the focus of the intervention, we need to evaluate the process of implementation more thoroughly (eg, which patients persist and which drop out) and the quality of communication. We observed that patients need more help with self-care than they received in the intervention settings, and online training and personal assistance might be necessary in cases of ICT-based care. A supportive health policy environment (and appropriate financing) is necessary to guarantee continuity after a pilot period. Successful diabetes management systems should integrate several functions to provide collaborative care and to meet the needs of patients and caregivers. Moreover, the shift from hospital to community centers or home care requires technology that integrates lifestyle and education functions for simultaneous group education and for encouraging self-care. Future research should be directed at the development of patient-centered technology personalized to specific needs and capacities. More rigorous methods are needed to measure the effects of an intervention on quality of life, well-being, and organizational issues such as cost effectiveness to make decisions on implementation and to encourage better care coordination. By means of usability tests and log files, patients’ needs for care and technology support can be measured, and test results can be linked to education and behavior changes [59]. By means of critical incidents techniques [60], the conditions that permit technology to be implemented successfully can be assessed. More transparency is needed in reporting economic evaluations. The costs included in the studies varied so that comparison of the reported savings is hardly possible, which is also demonstrated in a former review [57]. Cost effects should be studied with a clear perspective that reflects the purpose of the evaluation and the viewpoint of analysis (eg, cost-benefit, cost-effectiveness analysis).

We conclude that further assessment studies are needed to evaluate the contribution of ICT-based care to diabetes management. Future research should examine the potential of technology to enhance self-efficacy with the aim of making life worth living for someone with certain limitations, in cases where the disease is incurable. Technology can easily overstress the negative aspects of disease and illness because of the focus on collecting health data (eg, food intake). In the end, self-efficacy and social support are possibly the main conditions for changing health behavior [61].

## Multimedia Appendix

Extended Tables 3 and 5 (containing inclusion criteria and data gathering methods): Overview of videoconferencing and combined interventions

## Abbreviations

DM: diabetes mellitus
HbA_1c_: glycosylated hemoglobin
ICT: information and communication technology
RCT: randomized controlled trial
