# Supplementary material for: The Contribution of Teleconsultation and Videoconferencing to Diabetes Care: A Systematic Literature Review
Source: J Med Internet Res. 2007 Dec 14;9(5):e37. doi: 10.2196/jmir.9.5.e37 (PMC2270420; doi:10.2196/jmir.9.5.e37)
Supplement: Supplementary file 1 [file jmir_v9i5e37_app1.pdf]

Multimedia Appendix

The contribution of teleconsultation and videoconferencing to diabetes care: a systematic literature review

Upload the table as a word file under "supplementary file".

Verhoeven, F. MSc[1], Gemert-Pijnen, J.E. van PhD[1], Dijkstra, K. MSc[3], Nijland, N. MSc[2], Seydel, E.R. PhD[2], Steehouder, M.F. PhD[1]

[1]Faculty of Behavioral Sciences, Department of Technical and Professional Communication, University of Twente, Enschede, the Netherlands

[2]Faculty of Behavioral Sciences, Department of Psychology and Communication of Health

[3]Faculty of Behavioral Sciences, Department of Marketing Communication and Consumer Psychology, University of Twente, Enschede, the Netherlands

Corresponding author:

Fenne Verhoeven MSc

University of Twente

Faculty of Behavioral Sciences

Department of Technical and Professional Communication

P.O. Box 217

7500 AE Enschede

The Netherlands

Telephone: +31 53 489 4441

Fax: +31 53 489 4259

Email: f.verhoeven@utwente.nl

Table 3. Overview of teleconsultation interventions

Reference, country, year, and duration of intervention	Care setting and intervention	Study design and inclusion criteria	Data gathering methods	Reported findings
[13]<br><br>- Italy/Spain/Norway<br>- 2002<br>- 18 months, follow-up planned (duration unknown)	Secondary care. Bloodglucosemeter to send clinical data and lifestyle data (every seven to ten days) via telecommunication system (Internet / telephone line). Daily computer-generated feedback is provided, and if necessary, messages from physician (specialist in hospital) to advice patients. No details provided about feedback system and frequency of feedback.	- 4. Observational studies without control group: n=32<br>- Four conditions:<br>- *Verification phase: clinical evaluation (n=3)*<br>- *pilot clinical validation (n=12)*<br>- *Demonstration phase: intranet (n=6) and internet (n=11)*<br>- DM type 1<br>- Adolescents and adults<br>- Being well-compliant with therapy	- Logfiles<br>- Non-standardized questionnaire	a) Decreased HbA_{1c} (mean reduction of 1.23%, ns), one condition (intranet), NSD.<br>c) Patient-caregiver communication increased (messages sent from caregiver to patients and vice versa).<br>e) Reliable and helpful (caregiver) system, acceptable for patients.
[14]<br><br>- Italy/Spain/Germany<br>- 2003<br>- 12 months, follow-up unknown	Integrated care. Reflectometer and palmtop to transmit clinical data via multi access system (web access, telephone, interactive voice) to each agent involved in the care process; nurses, case managers, and specialists. Computer-generated feedback is provided via SMS or e-mail to patient and caregiver and educational messages are automatically sent to patients. Frequency of feedback not specified.	- 1. Experimental studies (RCT): n=106<br>- Two conditions:<br>- *Intervention: n=56 (subset randomized patients not reported)*<br>- *Control (usual care): n=50 (subset randomized not reported)*<br>- Randomization method not described; no details about comparability of group (except same clinical treatment)<br>- DM type (1,2 or gestational) not specified<br>- Being motivated to take part in the study<br>- Having internet access<br>- Having a cellular phone	- Validated questionnaires (World Health Organization Quality Of Life-Bref and Telemedicine Satisfaction Questionnaire)<br>- Logfiles	a) Decreased HbA_{1c} in I (from 8.31 to 7.59, $p<0.05$), in C (from 8.86 to 7.95, $p<0.05$), after 6 months, NSD between groups. Patients Randomized, decreased HbA_{1c} in I (from 8.24 to 7.44, $p<0.05$), in C (from 8.83 to 7.78, $p<0.05$), after 6 months, NSD.
[15]<br><br>- Germany<br>- 2002<br>- 4-8 months, follow-up unknown	Secondary care. Bloodglucosemeter to send clinical data via modem and telephone line to physician in diabetes centre. Personal feedback for proper dose adjustment by diabetes specialist via telephone advice. Frequency of feedback not specified.	- 1. Experimental studies (RCT): n=43<br>- Two conditions:<br>- *Intervention: n=27*<br>- *Control (usual care): n=16*<br>- Randomization by lots (2:1 in favour for telecare)<br>- Fairly good matching of groups<br>- DM type 1<br>- Having followed a structured diabetes education program<br>- Taking part in the intervention for at least 50%<br>- ≥ 4 doses of insulin/day	- Non-standardized questionnaire	a) Decreased HbA_{1c} in I (from 8.3 to 6.9 after 4 months, n=27, to 7.1 after 8 months, n=11), in C (from 8.0 to 7.0 after 4 months, n=16, to 6.8, n=10 after 8 months, NSD between groups.<br>e) System appeared easy to use, patients' feeling of security increased through availability of BG-data and the possibility of consulting a caregiver within minimal time, without the need to travel to the diabetes center.<br>f) Cost and time saving in I (saving consultation time although intensified contacts with caregiver), in I on caregiver's side patients time significantly increased.

Legend: 1 experimental study, 2 quasi-experimental study, 3 controlled observation, 3a cohort study, 3b case control study, 4 observational study without control group, 5 expert opinion.
a) clinical values; b) quality of life, c) interaction, d) self-care, e) usability of technology, f) cost reduction, g)transparency of guidelines, h) equity (availability of health care to everyone) NSD; not statistically significant difference, ns, statistically not significant; I=intervention; C=control group

Table 3. Overview of teleconsultation interventions (Continued)

Reference, country, year, and duration of intervention	Care setting and intervention	Study design and inclusion criteria	Data gathering methods	Reported findings
[16]<br><br>- Netherlands<br>- 1999<br>- 12 months, follow-up unknown	Primary/ secondary care. Electronic communication network, linking the physicians' computer-based patient records (GPs and interns in hospital) to enable electronic data interchange. System provides computer-generated prompts for physicians to deliver feedback (messages). Frequency of feedback not specified.	- 2. Quasi-experimental studies: n=275<br>- Two conditions:<br>- *Intervention: n=215*<br>- *Control (usual care): n=60*<br>- Intervention group consisted of patients from GPs with highest number of referred patients. Average age in intervention group higher, less DM1 patients than control group<br>- Having a high frequency of referrals to specialist	- Non-standardized questionnaire	a) Decreased HbA_{1c} in I (from 7.0 to 6.8, p<0.05) in C (from 6.6 to 6.5, p=0.52), NSD between groups.<br>c) Increased frequency of patient-caregiver communication (p<0.01), more complete information about patient care in I than in C.
[17]<br><br>- United States<br>- 2002<br>- 12 months, follow-up unknown	Secondary care. Health Hero iCare Desktop and Health Buddy appliance for daily monitoring of clinical data and educational reinforcement by case manager (profession not specified) in medical centre. System prompts to action if indicated by daily values. Personal feedback by telephone in case of alarming values.	- 3. Controlled observational studies (3a. cohort studies): n=338<br>- Two conditions:<br>- *Intervention: n=169*<br>- *Control: n=169 (cohort)*<br>- Cohort was representative of the general population in terms of ethnicity<br>- DM type not specified<br>- Economically disadvantaged<br>- Living in region<br>- Already having a caregiver taking part in the intervention<br>- Having telephone access<br>- Being able to handle the intervention technique<br>- Being able to read	- Logfiles<br>- Non-standardized questionnaire<br>- Validated questionnaire (SF-12)	b) Mean improvement in mental component (SF-12) after 6 months in I (p<0.0264) and in physical component after 6 months (p< 0.0518).<br>c) Increased satisfaction regarding communication with caregivers in I (from 88% of patients after three months to 95% at 1 year).<br>d) Better understanding of their medical condition (93% of patients), better able to manage their disease (93% of patients (after 1 year).<br>e) Ease of use increased over time (75% of patients after three months to 88% after 1 year).<br>f) Reduction of overall utilization and charges after 1 year; In I overall charges of $747 per patient per year; Inpatient admissions reduced 32% (p<0.07), emergency room encounters reduced 34% (p<0.06) post discharge care visits reduced 44% (p<0.028) outpatient visits reduced 49% (p<0.001).
[18]<br><br>- Denmark<br>- 2006<br>- 6 months, follow-up unknown	Integrated care. Website for transmission of blood glucose data entered by patient and reviewed by diabetes team (two diabetic nurses, one consultant doctor, one medical secretary and one dietician) and personal feedback by diabetes team by e-mail about diabetes regimen. Frequency of feedback not specified. Based on theory of patient-centeredness and the Bayesian model of carbohydrate metabolism.	- 4. Observational studies without control groups (case series): n=13<br>- Three conditions:<br>- *patients (n=3)*<br>- *health care professionals (n=5)*<br>- *health care professionals (n=5 focus group)*<br>- DM type 1<br>- Being able to handle the intervention technique	- Interviews validated by focusgroup	c) Patients experienced greater confidence and a more personal report with staff, after 6 months use of the system. E-mail facilitated a dialogue between patient and diabetes team.<br>d) Improved self-control (patients checked more often blood glucose), increased awareness of blood sugar regulations.<br>e) Diasnet caused changes in tasks and duties of the diabetes team, (required enhanced competences of nurse with regard to insulin dose adjustments), patients were dissatisfied with the feedback from staff.

Table 3. Overview of teleconsultation interventions (Continued)

Reference, country, year, and duration of intervention	Care setting and intervention	Study design and inclusion criteria	Data gathering methods	Reported findings
[19]<br><br>- United Kingdom<br>- 2005<br>- 9 months, follow-up unknown	Secondary care. Bloodglucosemonitor and telephone network for transmission of data and GPRS mobile phone to send data (daily) to diabetes nurse specialist in clinic. Real-time graphical phone-based feedback for the previous two weeks together with nurse-initiated support using a web-based graphical analysis of glucose self-monitoring results and personal feedback by phone in case of concerns. Frequency of feedback not specified. Based on theory of patient-centeredness.	- 1. Experimental studies (RCT): n=93<br>- Two conditions:<br>- *Intervention: n=47 (web-based graphical analysis, nurse initiated support*<br>- *Control (real-time graphical phone-based): n=46*<br>- Randomization (computer program); sex and psychiatric scores were evenly distributed between the randomized groups<br>- DM type 1; HbA_{1c} ≥8%-≥11%<br>- Age 18-30 years<br>- Insulin suboptimal	- Interviews	a) Decreased HbA_{1c} in I (from 9.2 to 8.6, p<0.001, after 9 months), in C (from 9.3 to 8.9, after 9 months, p<0.05), NSD between groups.<br>e) Difference in proportion of transmitted blood glucose results (40% more in I than in C), p<0.0001.
[20]<br><br>- Spain<br>- 2004<br>- 9 months, follow-up unknown	Care setting not specified. PC, web browser or a cellular phone with Wireless Application Protocol for transmission of clinical data. Automatic generated responses and personal feedback by physicians (not specified whether GPs or specialists in hospital) that could be read during patient's next online session. Frequency of feedback not specified.	- 4. Observational studies without control groups (case series): n=172<br>- Two conditions:<br>- *Case study: n=12*<br>- *Questionnaire: n=160 (135non-diabetic students, 25 diabetic patients)*<br>- DM type not specified<br>- Adults<br>- Age 18≤75 years<br>- Being able to handle the intervention technique<br>- Having internet access or having a cellular phone	- Non-standardized questionnaire<br>- Logfiles	d) Patients were satisfied with the continuity and self efficacy of care, lack of time was a drawback for 38% of them, 75% expressed a preference for sending data via a cellular phone (SMS).<br>e) Patients used the system every 2.0 days (SD 2.1), and doctors reviewed their patient data every 4.0 days (SD 3.9); the average number of visits to the website was 477 per month.
[21]<br><br>- Spain<br>- 2004<br>- 8 months, follow-up unknown	Care setting not specified. Patients send blood glucose levels and body weight to a server by SMS. Automatic server answers SMS each time data sent. Monthly haemoglobin results automatically sent to physicians (not specified whether GPs or specialists in hospital). Physicians can send messages to patients if necessary.	- 4. Observational studies without control groups (case series): n=23<br>- One condition<br>- DM type not specified<br>- Having a cellular phone	- Non-standardized questionnaire<br>- Logfiles	e) SMS provided a simple, fast, efficient and low-cost adjunct to the medical management of diabetes at a distance. In particular useful for age groups (elderly, teenagers) that are known to have difficulty in controlling diabetes well.<br>f) Total of 25 messages per month), €3.75 per month per patient

Table 3. Overview of teleconsultation interventions (Continued)

Reference, country, year, and duration of intervention	Care setting and intervention	Study design and inclusion criteria	Data gathering methods	Reported findings
[22]<br><br>- France<br>- 2006<br>- 6 months, follow-up unknown	Secondary care. Clinical data from patients' glucometers downloaded every two weeks to pharmacists' pc. Reinforced follow-up via fax mediated by the local pharmacist in contact with the specialist in the hospital (diabetologist). Diabetologist sends instruction to family by e-mail or phone within five days.	- 1. Experimental studies (RCT): n=100<br>- Two conditions:<br>- *Intervention: n=50*<br>- *Control (usual care): n=50*<br>- Randomization via computer generated sequence using block randomization with stratification by age<br>- Comparable groups Intervention and control (age, sex, HbA1c level, frequency of SBGM, type of insulin therapy program)<br>- DM type 1; HbA_{1c} ≥8%<br>- Age 8-17 years<br>- Diagnosed with DM > 1 year<br>- Living in region<br>- Having a scheduled doctor's appointment<br>- Pharmacist's agreement to participate in the study	- Logfiles	a) Decreased HbA_{1c} in I (from 9.3 to 9.27, p=0.59), in C (from 9.2 to 9.12, p=0.58), NSD between groups.<br>c) Caregivers responses to faxes was 81% (at third month), decreased to 50% (6 months).<br>d) Frequency of self blood glucose monitoring per day did not differ between groups at the end of the study (p=0.53).<br>e) 32% Faxes (out of the 100 expected) from families homes were received, due to technical problems.
[23]<br><br>- Spain<br>- 2002<br>- 12 months, follow-up expected	Secondary care. Clinical data from a bloodglucosemeter is sent (automatically or manually) from a patient unit to the medical workstation for physicians (diabetologist in hospital). System offers tools to collect, manage, view and interpret data and to exchange data and messages. Physicians personally answer patients' questions within 24 hours via system. Frequency of feedback not specified.	- 2. Quasi-experimental studies: n=10<br>- Two conditions: Intervention: n=5<br>- Control: n=5 (cross over design, switch half way through the trial)<br>- Both groups were comparable (intervention-time, inclusion criteria (inadequate metabolic control, DM duration of over 5 year)<br>- DM type 1<br>- Diagnosed with DM > 5 years<br>- Poor metabolic control	- Non-standardized questionnaire<br>- Logfiles<br>- Interviews	a) Decreased HbA_{1c} in I (from 8.4 to 7.9, p=0.053), increased in C (from 8.10 to 8.15, p=0.58), NSD between groups.<br>c) Patients transmitted 3524 blood glucose readings, 1649 daily insulin adjustments, 24 exercise reports and ten diet modifications. Electronic communication with caregivers was limited, a total of 63 text messages were sent by all patients. Caregivers sent 118 text messages to patients (feedback and therapy modifications). Caregivers performed more therapy changes in I than in C due to the possibility to assess patient's condition on a frequent basis.<br>d) Increased confidence in daily self management.<br>e) Patients found the system has high utility, despite several technical problems.

Table 3. Overview of teleconsultation interventions (Continued)

Reference, country, year, and duration of intervention	Care setting and intervention	Study design and inclusion criteria	Data gathering methods	Reported findings
[24]<br><br>- United States<br>- 2002<br>- 3 months, follow-up unknown	Primary care. E-mail for communicating disease management issues between Veterans Affairs Primary Caregiver and pharmacists. Personal feedback to patients via telephone by pharmacist. Frequency of feedback not specified.	- 2. Quasi-experimental studies: n=65<br>- Two conditions:<br>- *Intervention: n=30*<br>- *Control (usual care): n=35*<br>- Intervention: patients had a recent change made to their therapy to lower blood glucose levels<br>- Control: remaining patients of the 65<br>- Comparable HbA1c at baseline in two groups<br>- DM type 2; $HbA_{1c} \geq 9\%$<br>- Already having a caregiver taking part in the intervention<br>- No change in treatment for at least 60 days	- Logfiles	a) Decreased HbA_{1c} in I (from 10.0 to 8.2; p<0.001), in C (from 10.2 to 8.6, p<0.001), NSD between groups.<br>f) E-mail communication reduced the number of face to face and telephone consultations between care givers.<br>g) Clinical recommendations for altering diabetes care sent via e-mail to primary caregiver resulted in I in a significant reduction in HbA_{1c}.
[25]<br><br>- Spain<br>- 2006<br>- 12 months, follow-up unknown	Secondary care. Data from glucometer and vocal messages concerning insulin doses and events are sent via modem (twice a week) to diabetes team (in hospital, members of diabetes team not specified). Diabetes team provides personal feedback. No details provided about form and frequency of feedback.	- 1. Experimental studies (RCT): n=30<br>- Two conditions:<br>- *Intervention: n=18*<br>- *Control (usual care): n=15*<br>- Randomization via random variable generator. Baseline data (A1C, BM, weight, insulin, DM) and characteristics (age, sex, daily activities) were comparable in two groups<br>- DM type 1; $HbA_{1c} \geq 8\%$<br>- Age 18-50 years<br>- Diagnosed with DM > 2 years<br>- ≥ 3 doses of insulin/day	- Interviews<br>- Focusgroups<br>- Non-standardized questionnaire<br>- Logfiles<br>- Validated questionnaires (SF-12 + Diabetes Quality of Life + DQK2-test + Telemedicine Satisfaction Questionnaire)	a) Decreased HbA_{1c} in I (from 8.4 to 7.6), in C (from 8.9 to 7.6), after 12 months, NSD between groups<br>b) General health status did not change in groups (SF-12) , quality of life improved in I (ns) and C (p<0.05); significant increase in knowledge in I (p<0.05) and in C (p<0.05).<br>d) 80% Of patients reported that appointments in I did not interfere with daily life; in C 100% of patients reported daily interference with outpatients appointments.<br>f) Time and costs saved by patients. Costs were lower (length of appointment 0.25h in I versus 0.5h in C). But 30% of the diabetes team and patient appointments were longer than expected due to technical problems (0.25h versus 1h).
[26]<br><br>- South Korea<br>- 2006<br>- 12 weeks, follow-up unknown	Tertiary care. Clinical data is entered daily in system via website or cellular phone (SMS). Automatic feedback (reminder) is generated in case patient has not forwarded data for more than a week. Personal feedback provided weekly by nurse in tertiary care hospital via SMS, telephone or Internet.	- 4. Observational studies without control groups (Before-and-after design): n=42<br>- One condition<br>- DM type 2<br>- Being able to handle the intervention technique<br>- Having internet access<br>- Having a cellular phone	- Logfiles<br>- Interviews<br>- Visual Analog Scale	a) Patients had a mean decrease of 28.6 mg/dL in fasting plasma glucose p=0.006) and 78.4mg/dL in 2-hour postprandial blood sugar levels, p=0.003).<br>d) Mean increase in care satisfaction score in I (from 68.6 to 79.5, p=0.03).

Table 3. Overview of teleconsultation interventions (Continued)

Reference, country, year, and duration of intervention	Care setting and intervention	Study design and inclusion criteria	Data gathering methods	Reported findings
[27]<br><br>- Italy<br>- 2006<br>- 56 weeks, follow-up unknown	Care setting not specified. Blood glucose data from a glucometer is sent via internet or telephone to the system. Data is automatically analyzed in order to detect metabolic alterations and, if necessary, generates alarms. If necessary, physician (not specified whether GP or specialist in hospital) responds and a message is automatically sent to patient by e-mail or SMS. Frequency of feedback not specified. Based on a general model for the coordination of care (Chronic Care model).	- 1. Experimental studies (RCT): n=56<br>- Two conditions:<br>- *Intervention: n=30*<br>- *Control (usual care): n=26*<br>- Randomization; no details. Both groups were comparable (age, treatment)<br>- DM type not specified	- Logfiles<br>- Cognitive walkthrough<br>- Non-standardized questionnaire	a) Location 1, decreased HbA_{1c} in I (from 8.52 to 8.30), p<0.05), in C (from 8.97 to 8.82), NSD between groups. Location 2, decreased HbA_{1c} in I (from 8.40 to 7.75) and in C (from 10.15 to 9.28), after 12 months, NSD between groups.<br>c) Patients transferred 20,000 BGL readings and 2000 insulin doses (56 weeks, over 2 locations), the frequency of service usage and quantity of data collected were considered satisfactory.<br>e) Overall usability perception was high (TSQ) especially in adult patients.
[28]<br><br>- United States<br>- 2005<br>- At least 24 months, follow-up unknown	Primary care setting. Blood glucose data from glucometers sent to the diabetes team weekly (team composition not specified) and nurses of the children's medical care service clinic. Feedback is provided during a clinical, face-to-face session. Online education for school personnel, families, and caregivers is provided on a website. No details provided about form and frequency of feedback.	- 4. Observational studies (case series): n=74<br>- Four conditions:<br>- *Patients: n=44*<br>- *Caregivers: n=6*<br>- *Case managers: n=6*<br>- *School nurses: n=18*<br>- DM type 1<br>- Paediatric patients<br>- Without adequate insurance<br>- Chronically ill	- Non-standardized questionnaire	c) Improved patient-caregiver communication for patients in a remote area. Use of website by nurses increased substantially when it was approved for three contact hours of continuing education.<br>d) 40% Of patients completed educational modules on the website.<br>e) Users (patients, family, and school nurses) expressed satisfaction with technology.<br>g) Compliance with school health plans improved compared with baseline.
[29]<br><br>- Australia<br>- 2002<br>- 6 weeks, no follow-up	Care setting not specified. Internet-based diabetes management systems (my Diabetes, LifeMasters) for evaluation of diabetes management. No details about form and frequency of feedback. Based on the push-pull model for retrieving and seeking information.	- 5. Expert opinion based on consensus: n=5 (1 caregiver, 3 diabetes patients,1 expert)<br>- One condition<br>- DM type not specified	- Checklist (non-standardized)	e) LifeMaster appeared successful in integrating the health caregiver in diabetes management; My Diabetes is effective in providing a communication channel for community creation. LifeMaster appeared a more complete system than MyDiabetes (monitoring, personalization, communication, information, technology).

Table 3. Overview of teleconsultation interventions (Continued)

Reference, country, year, and duration of intervention	Care setting and intervention	Study design and inclusion criteria	Data gathering methods	Reported findings
[30]<br>- United States<br>- 3 months, follow-up unknown	Primary care. Internet-based, diabetes self-management and peer support intervention (chat room). The Diabetes network was designed to complement medical treatment by providing personalized lifestyle interventions, and social support via an internet based program accessible from patients' home. Simplified computers and training were used. Online blood glucose tracking, twice weekly patient-physician (primary care provider) contact (questions), post messages on forum (real time chat discussion). Personal dietary advice by primary care provider via website, forum.	- 1. Experimental studies (RCT): n=133<br>- Four conditions:<br>- *Information only group: n=33*<br>- *Peer support group: n=30*<br>- *Personal self management condition: n=37*<br>- *Combined condition of the three above: n=33*<br>- Randomization by presence of absence of each of the components (peer support, personalized self management). Groups were comparable (sex, education, age, years diagnosed).<br>- DM type 2<br>- Age 40-75 years<br>- Living in region; Having the intention to stay in the region the next year<br>- Having telephone access<br>- Being able to read and write<br>- Having no Internet access	- Logfiles<br>- Validated questionnaires (Diabetes Quality Of Life + SF-12 + Depression Scale (CES-D)	a) Decreased HbA_{1c} in I (PSMCC) from 7.75 to 7.73), in I (PSC) from 7.64 to 7.59, in I (CC) from 7.46 to 7.28) in C increased (from 7.20 to 7.37) after 3 months.<br>Overall improvement dietary behavior (reduction of fat intake, improved dietary practices) in 4 conditions, but no significant between-condition differences.<br>b) Slight improvements in quality of life (psychological well-being SF-12) in 4 conditions, especially for PSMCC and CC.<br>c) Two support conditions (PSC,CC) generated significantly more logons (Ms 61 and 70, resp. for PSC and CC and M=40 (PSMCC), and M=25 in IOC; p<0.02).
[31]<br>- United States<br>- 2005<br>- 12 months, follow-up unknown	Primary care. Clinical data from glucometers are sent three times a week via Internet to a website. Web-based care management group received a notebook, glucose and blood pressure monitoring devices, and access to a care management website. The Website provides educational modules, accepting uploads from monitoring devices and an internal messaging system for patients to communicate with the care manager. Automatic feedback is provided if patients have not forwarded data in two weeks. Care manager contacts patients by phone, diabetes nurse communicates with patients about education using the internal messaging system. The care manager responded to queries within 1 working day during office hours.	- 1. Experimental studies (RCT): n=104<br>- Two conditions:<br>- *Intervention: n=52*<br>- *Control (usual care): n=52*<br>- Randomization, no details. Both groups were comparable (age, sex, education, metabolic values)<br>- DM type not specified; $HbA_{1c} \geq 9\%$<br>- Age >18 years<br>- Already having a caregiver taking part in the intervention	- Logfiles	a) Significant decrease in HbA_{1c} in I and C (p<0.001). A greater decline in A1C over time (12 months) in I (10.0, -1.6%) and in C (9.9, -1.2%), p<0.05. Individuals who persisted with website usage (at least one website log-in every 3 months, p<0.05) had a greater improvement in HbA_{1c} than usual care.<br>HDL cholesterol rose and triglycerides fell in the web based group (p<0.05).<br>d) Regular data uploads (p<0.02) were more likely to achieve and maintain reductions in HbA_{1c}.

Table 3. Overview of teleconsultation interventions (Continued)

Reference, country, year, and duration of intervention	Care setting and intervention	Study design and inclusion criteria	Data gathering methods	Reported findings
[32]<br><br>- United States<br>- 2004<br>- 3 months, follow-up unknown	Primary care. Web-based disease management programme based on an interactive electronic medical record and secure e-mail system. System contains My Upload Meter to automatically upload clinical data daily sent and Diabetes Daily Diary educational website. Automatically generated clinical reminder, e-mail response every weekday (by nurse practitioner in primary care internal medicine clinic). Based on a general model for the coordination of care such as the Chronic Care model.	- 4. Observational studies (before-and-after design): n=9<br>- One condition<br>- DM type 2<br>- The population had to ensure a range of patient characteristics including age, sex, years with diabetes, and distance from the clinic	- Focus group<br>- Interviews	c) If expectations were not met, participants felt their concerns were less valued, and they felt more isolated from their caregiver.<br>d) Participants felt safer having real-time access to their personal health information. Feeling more able to manage diabetes by means of seeing laboratory results in the live record at home.<br>e) Frustration with unmet expectations; when program did not work as expected (technical failures).
[33]<br><br>- Netherlands<br>- 2001<br>- duration not specified, follow-up unknown	Primary/secondary care. Shared-care project whereby all examinations, which take place every three months and are performed by the GP, follow standardized procedures. Results are emailed to the diabetologist and laboratory results are automatically sent to both GP and diabetologist. Feedback by post from diabetologist to GP.	- 4. Observational studies (case series): n=594<br>- Three conditions:<br>- *Patients treated in project: n=336*<br>- *Patients treated by GP: n=225*<br>- *Patients treated in outpatient clinic: n=33*<br>- DM type 2<br>- Already having a caregiver taking part in the intervention	- Non-standardized questionnaire<br>- Interviews	a) Decreased HbA_{1c} in UDP (from 7.8 to 6.8, p<0.0001), mean inclusion duration 3.2 years. Lipid profiles improved in I (Plasma cholesterol decreased (from 6.1 to 5.9, p<0.0001), plasma triglyceride decreased (from 1.9 to 1.7), p<0.0001) and diastolic blood pressure 86 to 83 (p<0.001).<br>d) Data records of UDP cohort were most complete compared to other groups.<br>e) GPs intend to continue participating in UDP despite shared care took more time.<br>g) Standardized data transfer (protocol driven) between GP, diabetologist, laboratory established an effective infrastructure for shared diabetes care.
[34]<br><br>- China<br>- 2001<br>- 12 weeks, follow-up unknown	Secondary care. Dietary and clinical data is recorded in hand-held computer and sent twice a week via a modem to the diabetes team of a hospital diabetes clinic (composition of diabetes team not specified). System generates automatic feedback about content of food.	- 2. Quasi-experimental studies: n=19<br>- Two conditions:<br>- *Intervention: n=10*<br>- *Control: n=9*<br>- Each group used the DSM three months, served as the control group for another three months (cross over design); comparable groups<br>- DM type not specified<br>- Already having a caregiver taking part in the intervention	- Non-standardized questionnaire<br>- Interviews	a) Decreased HbA_{1c} in I (8.56 to 7.55 after treatment and 7.84 end of project 12 weeks) , in C (8.81 to 8.76 after treatment, and 8.40 after end project12 weeks). Mean difference was 0.825, p<0.019, n=19)<br>e) The DSM was acceptable; 95% found it easy to use, 63% found it useful.

Table 5. Overview of videoconferencing and combined interventions

Reference, country, year, and duration of intervention	Care setting and intervention	Study design and inclusion criteria	Data gathering techniques	Reported findings
[35]<br><br>- Austria<br>- 2002<br>- 12 months, follow-up unknown	Primary/ secondary care: Patient and GP consult specialist in diabetes centre via PC fitted with videoconferencing card connected to a single ISDN line. Personal feedback (diabetologist) on therapy change during video session. Based on organizational learning theory.	- 4. Observational studies (before-and-after study): n=154<br>- One condition<br>- DM type 2<br>- Diagnosed with DM > 1 year<br>- Being treated > 1 year	- Semi-structured interviews	a) Decreased HbA_{1c} (from 8.1 to 7.8, p<0.05, after 12 months), systolic blood pressure (from 156.0 to 148.0 mmHg, p<0.0005), diastolic blood pressure (from 88.0 to 83.0 mmHg, p<0.0005) GPs measured late complications and metabolic parameters more frequently during the project than they did before.<br>e) Technical quality of therapeutic counseling via videoconferencing was sufficient, good enough to evaluate the clinical course of foot ulcer; duration of interview via videoconferencing with patients was on average 12 min (range 4-23).<br>f) Reduction of hospital admission from 12 before Intervention to 7 (during 1 year); duration of hospitalization for whole patient group for treatment of acute complications reduced (from 110 to 68 days per year).
[36]<br><br>- China<br>- 2005<br>- 8 weeks, no follow-up	Secondary care: Patients in a community centre and specialist in hospital connected via a large screen television in the centre and a digital camera for better visualization of skin and wound condition and internet protocol networking videoconferencing units with televisions. Educational sessions (caregiver not specified) regarding diet and ideal body weight, foot care, glucose monitoring and exercise prescription. No details provided about form and frequency of feedback. Based on a model of service delivery using the group setting for education regarding disease management of elderly people with diabetes.	- 4. Observational studies (before-and-after study): n=22<br>- One condition<br>- DM type 2<br>- Age > 60 years<br>- Limiting mobility or daily living	- Non-standardized questionnaire<br>- Focus group<br>- Logfiles<br>- Validated questionnaires (Diabetes Quality Of Life+ SF-36 + Diabetes Knowledge Assessment)	a) Reduction in total calorie intake (energy p<0.000, carbohydrates p<0.002, protein p<0.039, fat p<0.001) and body mass index (p<0.005).<br>b) Improvement in disease specific and generic measures of quality of life (SF-36, physical p<0.000, general health p<0.001, vitality p<0.005, social p<0.013, emotional, p<0.019; DQOL, p<0.000).<br>d) Improved disease-knowledge (DKN), (mean score from 7.91 to 13.05), better diabetes control (measured by 2-hr hemastix).<br>c/e) Patients accepted videoconferencing, preferred face to face interaction, staff found system easy to use.
[37]<br><br>- Norway<br>- 2005<br>- duration not specified, follow-up unknown	Secondary care: Treatment of diabetic foot ulcers whereby a nurse goes to the patients' house with videophone and laptop and consults physician (specialist in hospital). An online ulcer record system is available capable of notifying the physician by SMS messages. Feedback from physician via videophone.	- 4. Observational studies without control groups (case series): n=20<br>- Two conditions:<br>- *Workshops: n=15*<br>- *Pilot test: n=5*<br>- DM type not specified	- Focus group<br>- Logfiles<br>- Interviews<br>- Observations	e) Staff and patients found equipment easy to use.<br>f) Patients saved time (no travel to hospital, no waiting time).<br>g) Shared documentation enhanced treatment (coordination).

Legend: 1 experimental study, 2 quasi-experimental study, 3 controlled observation, 3a cohort study, 3b case control study, 4 observational study without control group, 5 expert opinion.
a) clinical values; b) quality of life, c) interaction, d) self-care, e) usability of technology, f) cost reduction, g)transparency of guidelines, h) equity (availability of health care to everyone) NSD; not statistically significant difference, ns, statistically not significant; I=intervention; C=control group

Table 5. Overview of videoconferencing and combined interventions (Continued)

Reference, country, year, and duration of intervention	Care setting and intervention	Study design and inclusion criteria	Data gathering techniques	Reported findings
[38]<br><br>- United States<br>- 2001<br>- 24 months, follow-up unknown	Primary care: Video visits in addition to skilled nursing visits (Visiting Nurse Association). Patient station in home with camera with close-up lens. Patient and clinical station linked together over ordinary telephone lines via standard modem. No details provided about form and frequency of feedback.	- 1. Experimental studies (RCT): n=171<br>- Two conditions:<br>- *Intervention (1 video visit, 1 home visit, 1 video visit): n=86*<br>- *Control (skilled nursing home visits only): n=85*<br>- Randomization, no details. Groups were comparable (sex, average age, mean diabetes severity score, mean number of co-morbidities)<br>- DM type not specified<br>- Already having a caregiver taking part in the intervention	- Interviews<br>- Cost Economic analysis<br>- Validated questionnaire (SF-36)	f) Cost savings without compromising quality, videoconferencing has the potential to provide the same number of patient encounters at lower costs, financially benefit increases as the duration of the patient care episode increases.<br>Fewer videoconferencing patients required recertification after 60 days in I compared to C (23% versus 25.6%, p<0.001), 63.7% of videoconferencing patients were discharged to home care to 39% of C group, p<0.001. 28% of C-group were hospitalized during 60 days compared to 10% of videoconferencing patients, p<0.05.
[39]<br><br>- United States<br>- 2003<br>- 3 months, in case of success, follow-up	Secondary care: Patients report blood sugar levels, injections, and food intake daily either by telephone, videophone (analogue videophone connected to a television) or e-mail. Psychological staff provides advice on changing and maintaining behavior (phone, videophone or e-mail). Diabetes nurse ensures medical needs.	- 4. Observational studies (case series): n=5<br>- Three conditions:<br>- *Telephone intervention: n=3*<br>- *Videophone intervention: n=1*<br>- *E-mail intervention: n=1*<br>- DM type not specified<br>- Adolescents<br>- Already having a caregiver taking part in the intervention<br>- Extremely poor metabolic control<br>- Psychiatric disorders	- Interviews	a) Decrease of HbA_{1c} (for each child, from 9.7 to 8.5; from 8.7 to 7.1; from 13 to 6.1, from 10.2 to 9.4; from 10.9 to 7.9), after 3 months.<br>d) Better self-control (managing the sending of blood sugar), no hospitalizations, no school absences.
[40]<br><br>- Canada<br>- 2002<br>- 3 months, follow-up unknown	Secondary care: Video visits whereby patient at home communicates with physician in hospital. Equipment not specified. No details provided about form and frequency of feedback. Based on Donabedian's principle of quality of care.	- 4. Observational studies without control groups (case series): n=25<br>- Four conditions:<br>- *Patient group: n=8*<br>- *Nurse group: n=13*<br>- *Physicians: n=7*<br>- *Managers: n=7*<br>- DM as a primary or secondary diagnosis<br>- Adult home care clients<br>- Already having a caregiver taking part in the intervention<br>- Not cognitively impaired	- Focus group<br>- Interviews	e) Patients and managers identified a higher degree of readiness for videoconferencing, patients because of the potential to support independence in their homes, managers because of efficiency of the system. Patients wanted to maintain their level of health, but with minimum intrusiveness, caregivers were more interested in measurable clinical outcomes (blood pressure, glucose), managers focused on cost-effectiveness.

Table 5. Overview of videoconferencing and combined interventions (Continued)

Reference, country, year, and duration of intervention	Care setting and intervention	Study design and inclusion criteria	Data gathering techniques	Reported findings
[41]<br><br>- United States<br>- 2003<br>- 3 months, follow-up unknown	Primary care: Patients at remote telemedicine site connected to nurse educator and dietician in diabetes centre through videoconferencing. Both sites equipped with PC, digital camera and a conference system. Three one-on-one monthly educational sessions (nurse and educator). Feedback given by nurse educator and dietician during sessions.	- Experimental studies (RCT): n=46<br>- Two conditions:<br>- *Intervention: n=24*<br>- *Control (education in person): n=22*<br>- Randomization via random permuted blocks. Groups were comparable (age, sex, BM, duration diabetes)<br>- DM type not specified<br>- Age 18≤75 years<br>- Already having a caregiver taking part in the intervention<br>- Not having had diabetes education for at least 1 year<br>- Being able to read, understand, and sign the consent document	- Validated questionnaires (Diabetes Quality Of Life + Telemedicine Satisfaction Questionnaire + Diabetes Treatment Satisfaction Questionnaire + SF-36 + Appraisal of Diabetes Scale + Problem Areas In Diabetes-scale)	a) Decrease of HbA_{1c} in I (from 8.7 to 7.8, p<0.001) in C (from 8.6 to 7.6, p<0.001), after 3 months,NSD between groups.<br>b) Reduced diabetes related stress was observed in I and C, p<0.007, NSD between groups.<br>d) More positive appraisal of their diabetes (p<0.05) in both groups.<br>e) Most patients who received videoconferencing felt comfortable with videoconferencing and found it very convenient; overall satisfaction was high (score 4.3 of 5). Satisfaction with treatment increased in both groups (p<0.001), NSD between groups.
[42]<br><br>- United States<br>- 2005<br>- at least 24 months, follow-up unknown	Secondary care: Nurse and patient in clinic consult physician in hospital via videoconferencing equipment and hand camera, semi-monthly. An educational website covers the basics of diabetes care. No details provided about form and frequency of feedback.	- 4. Observational studies (before-and-after study): n=44<br>- One condition<br>- DM type not specified<br>- Paediatric patients	- Non-standardized questionnaire<br>- Interviews<br>- Cost Economic analysis	e) Over 90% of patients and family members expressed satisfaction with videoconferencing.<br>f) Reduced hospitalizations (before Intervention, on average 13 a year (47 days), after Intervention 3.5 a year (5.5 days). Reduced emergency department visits (from 8 to 2.5 a year). The visit interval decreased from 149 to 89 days as the bi-weekly telemedicine clinics replaced quarterly clinics.<br>h) Improved access to specialized health care via videoconferencing (underserved area), in combination with online education improved health status.
[43]<br><br>- Australia<br>- 2003<br>- 28 months, follow-up unknown	Secondary care: Video consultation between patient (groups) at regional centre and paediatric specialist in hospital used in three ways: (1) routine specialist clinics via videoconference using PC with videoconferencing equipment, digital camera and ISDN line, (2) ad hoc patient consultations at time of urgent clinical need, and (3) education to staff and patients throughout the state. Personal feedback by specialist in hospital to patient and staff during video session. Frequency of feedback not specified.	- 4. Observational studies (case series): n=170<br>- Three conditions:<br>- *Routine consultation: n=135*<br>- *Complication consultations with 1 patient: n=25*<br>- *Education sessions: n=10*<br>- DM type not specified<br>- Paediatric patients	- Logfiles	f) Reduced travel time for specialists hospital staff (by conducting clinics via videoconferencing); while maintaining patients' contact.<br>h) Improved access to specialist services (telepaediatric) from rural and remote areas.

Table 5. Overview of videoconferencing and combined interventions (Continued)

Reference, country, year, and duration of intervention	Care setting and intervention	Study design and inclusion criteria	Data gathering techniques	Reported findings
[44]<br><br>- United States<br>- 2000<br>- 3 months, follow-up unknown	Primary care: Monitoring metabolic values and dietary behavior from patients' home unit to primary care clinic, family practice or internal medicine at the medical centre. Weekly patient-nurse consultation through videophone over a telephone line to discuss metabolic values and dietary behavior. E-mail contact maintained between case manager, specialist, and the family practitioner. Nurse case manager provides advice once a week during session; primary care physician is contacted once a month (for advice).	- 1. Experimental studies (RCT): n=28<br>- Two conditions:<br>- *Intervention: n=15*<br>- *Control (no information available about control group): n=13*<br>- Randomization (stratified based on age, gender, microalbumin, creatinine, A1C)<br>- Comparable groups<br>- DM type 2; $HbA_{1c} \geq 8\%$<br>- Age >18 years	- Non-standardized questionnaire<br>- Validated questionnaires (Diabetes Quality Of Life, SF-36)	a) Decreased HbA_{1c} in I (from 9.5 to 8.2, $p<0.05$), in C (from 9.5 to 8.6), after 3 months, NSD between groups. Mean weight reduction (4%).
[45]<br><br>- United States<br>- 2004<br>- 12 months, follow-up unknown	Secondary care: Self-management therapy video consultation (on nutrition) between patient at home and specialist in hospital. The telediabetes program had been in operation for 10 years. Used equipment not specified. Registered nurse conducts educational session with patient by videoconferencing.	- 4. Observational studies (case series): n=60<br>- One condition<br>- DM type not specified<br>- Already having a caregiver taking part in the intervention	- Non-standardized questionnaire<br>- Interviews	c) Sustainability of the telediabetes program depends on a feedback system; the effectiveness of the process depends on an interactive, ongoing collaboration between patient and caregiver.<br>f) Reduced travel time for patients and caregivers.<br>g) Administration took a long term view of the value of telemedicine service, service delivery followed national diabetes standards and a well defined cycle of care within a long-term quality improvement program and consistent education program resulted in sustainable diabetes care.<br>h) The system provided access to specialized health care to remote areas.
[46]<br><br>- United States<br>- 2004<br>- 12 weeks, follow-up unknown	Secondary care: Physician and physical therapist in hospital connected with patient and nurse in medical centre for the treatment of diabetic foot ulcers. Both equipped with a video conferencing unit and a television monitor. The hospital has a handheld camera for real-time transmission of close-up images of the foot and a document camera for real-time transmission of foot X-ray images. Personal feedback by nurse during weekly session.	- 2. Quasi-experimental studies: n=140<br>- Two conditions:<br>- *Intervention: n=20*<br>- *Control (face to face foot program): n=12*<br>- Comparable groups (age, wound condition)<br>- DM type not specified	- Interviews	a) No difference in I and C in the average forefoot ulcer healing time, the percentage of ulcers healed in 12 weeks, and the adjusted healing time ratio.<br>e) Equipment appeared easy to use and provided clear viewing of foot lesions and X-rays. Patients appeared well satisfied with use of technology.<br>f) Patients saved travel time.<br>h) Patients appreciated the convenience of being treated at their local facility, had more access to specialized care).

Table 5. Overview of videoconferencing and combined interventions (Continued)

Reference, country, year, and duration of intervention	Care setting and intervention	Study design and inclusion criteria	Data gathering techniques	Reported findings
[47]<br><br>- China<br>- 2002<br>- 18 weeks, follow-up unknown	Primary care: Education in small patient groups in health centre, given by nurse in diabetic centre through videoconferencing equipment connected by a local area network. Four sessions, each lasting 2 hours. Personal feedback (from diabetes nurse) during sessions.	- 4. Observational studies (case series): n=41<br>- One condition<br>- DM type 2; $HbA_{1c} \geq 7\%$<br>- Age 40≤70 years<br>- Chinese nationality<br>- Being recently referred to diabetes clinic<br>- Being on a diet<br>- No comorbidities	- Non-standardized questionnaire<br>- Validated questionnaire (Telemedicine Satisfaction Questionnaire	c) Videoconferencing enabled community nurses in primary care to link with nurse specialist in diabetes centre; to provide diabetes education in small groups.<br>d) Diabetes education conducted via videoconferencing was highly acceptable (mean total core (TSQ) was 61.9 of 75).<br>e) Significant positive correlation between age and satisfaction (r=0.39), the older the patient the higher the level of satisfaction with videoconferencing and with caregiver; no relationship between satisfaction with videoconferencing and with baseline HbA_{1c} level. Lack of a perceived need to have assistance while using equipment and the perceived ability of videoconferencing to meet health-care needs were most important predictors of satisfaction (accounted for 82% of the variance in satisfaction).<br>f) Patients saved travel time and waiting time.
[48]<br><br>- United States<br>- 2005<br>- 24 months, follow-up unknown	Primary care: Intervention consists of three parts:<br>1. Handheld in-home messaging device (Health Buddy) with disease management dialogues: Patients answer a daily series of questions and the care coordination staff of Veterans Health Administration (staff composition not specified) reviews responses daily to determine the level of risk for health care emergencies.<br>2. Telemonitoring with two-way audio-video connectivity that allowed for weekly monitoring of glucose and vital signs<br>3. Videophone with two-way audio-video connectivity, not including biometric monitoring. Patients followed up on a weekly basis for biometric info. Care coordinator reviews data daily to determine risks. Based on Wagner's Chronic Care Model.	- 4. Observational studies (before-and-after study): n=445<br>- One condition<br>- DM type not specified<br>- Veterans<br>- Complex medication conditions<br>- At high risk of expensive, multiple inpatient and outpatient service visits	- Retrospective analysis of costs<br>- Validated questionnaire (SF-36)	b) Significant improvement in health-related quality of life during 1 year (role-physical functioning p=0.02, bodily pain, p=0.005 and social functioning, p<0.05, SF-36).<br>e) Patients found equipment easy to use (>95% of patients).<br>f) Reduction in proportion of patients who were hospitalized (50% reduction, p<0.0.001), emergency room visits (11% reduction p<0.04), reduction in average number of bed days of care (decreased an average of 3.0 days, p<0.0001). Patients were 35% more likely to have had one or more need-based primary care clinic visits (p=0.0004).

Table 5. Overview of videoconferencing and combined interventions (Continued)

Reference, country, year, and duration of intervention	Care setting and intervention	Study design and inclusion criteria	Data gathering techniques	Reported findings
[49]<br><br>- United States<br>- 2005<br>- 24 months, follow-up unknown	Primary care: See [48]. Comparison of weekly monitoring with care coordinator versus daily monitoring with home message system. Personal feedback if necessary: Caregiver calls patient or facilitates an appointment. Instant camera with grid film for following diabetic wounds for aggressive wound managing (weekly monitored group targeted patients with active diabetic wounds). Patient takes two pictures of wounds, and mails it to care coordinator. Care coordinator reviews data daily to determine risks. The daily monitored group consists of diabetes that had wounds that required careful monitoring.	- 2. Quasi-experimental studies: n=297<br>- Two conditions (two different monitoring intensities):<br>- *Weekly monitoring, intensively monitored: n=197*<br>- *Daily monitoring, less intensively: n=100*<br>- In weekly monitored group patients were younger, in daily program more patients were married, both groups were comparable in clinical and sociodemographic characteristics<br>- DM type not specified<br>- Veterans<br>- Non-institutionalized<br>- Two hospitalizations<br>- At high risk of expensive, multiple inpatient and outpatient service visits<br>- Complex medication conditions	- Retrospective analysis of costs<br>- Validated questionnaire (SF-36)	a) Decreased HbA_{1c} in I (weekly monitoring) (from 8.3 to 8.1, p=0.22) and in C (daily monitoring), (from 8.7 to 8.8, p=0.78), after 24 months, NSD between groups. Adjusted mean values HbA_{1c} in I (weekly monitoring) (from 8.1 to 7.8, p=0.20) and in C (daily monitoring), (from 8.6 to 8.7, p=0.79), after 24 months, NSD between groups<br>f) Proportion of one or more hospital admissions decreased in daily monitoring group (77 to 43, p<0.01), and increased in the weekly monitoring group (73 to 106, p<0.01). The change in the average number of hospital bed days was eight days lower in daily monitored group than in the weekly monitored group (p<0.0001). Unscheduled primary care clinic visits were lower in the daily monitoring group (67 to 16) than in the weekly monitoring group (108 to 116), (significant difference between the two groups, p<0.01).
[50]<br><br>- United States<br>- 2005<br>- 12 months, follow-up unknown	Primary care: Patient-centered care coordination/home telehealth program, based on Wagner's Chronic Care Model, provides self-management and decision support, via electronic reminders and care coordinator. The system used an in home dialogue box (via patients' cell phone) to answer questions about health status, Answers were daily sent to internet to care coordinator who response in case of alarming values. Besides a two-way audio video connectivity and videophone were used (see [48]).	- 3. Controlled observational studies (3a. cohort studies): n=800<br>- Two conditions:<br>- *Intervention: n=400*<br>- *Control (no intervention): n=400*<br>- Propensity scores were used to improve the match between I and C<br>- A difference in difference approach was used to measure the effects of the intervention on service use<br>- DM type not specified<br>- Veterans<br>- At high risk of expensive, multiple inpatient and outpatient service visits<br>- Complex medication conditions<br>- Having telephone access	- Retrospective analysis of costs	f) Significant difference between I and C in need-based primary care visits, increasing in I (7.6%) and decreasing in C (12%), (p<0.01). The likelihood of 1 or more emergency department visits decreased in I and C (significant differences between groups, p<0.0001). I-group had a relative lower likelihood of having 1 or more hospitalizations than patients in the control group (control for HbA_{1c}, ns difference between I and C).<br>h) Increase in access to care (I)

Table 5. Overview of videoconferencing and combined interventions (Continued)

Reference, country, year, and duration of intervention	Care setting and intervention	Study design and inclusion criteria	Data gathering techniques	Reported findings
[51]<br><br>- United States<br>- 2005<br>- 5 months, follow-up unknown	Primary care: Monitoring blood glucose via a home telemedicine unit coupled with care management delivered from the diabetes centres at two hospital-based hubs. Patients can upload monitoring data, send secure e-mail, access an educational website, and use two-way video / voice conferencing. No details provided about form and frequency of feedback.	- 4. Observational studies (case series): n=5<br>- One condition<br>- DM type not specified	- Logfiles<br>- Visit logs<br>- Interviews	e) Technology related problems (telecommunication; connectivity) were the primary cause of installation difficulties (at patients' home). Patient education and training are the most critical success factors. Patient education and training accounted two third of the in-home time for installation of equipment. Nurse installers are patient centric rather than technology centric patient centric rather than technology centric
